# Supplementary material for: Effect of dietary omega-3 fatty acid supplementation on frailty-related phenotypes in older adults: a systematic review and meta-analysis protocol
Source: BMJ Open. 2018 May 17;8(5):e021344. doi: 10.1136/bmjopen-2017-021344 (PMC5961576; doi:10.1136/bmjopen-2017-021344)
Supplement: Supplementary file 1 [file bmjopen-2017-021344supp001.pdf]

Supplementary File: Search Strategy

Study population terms:

- Population, target condition and outcomes: Older people and frailty

('aged' OR 'old' OR 'age-old' OR 'elder' OR 'senior' OR 'functionally impaired' OR 'frail' OR 'exp frail elderly' OR 'ageing' OR 'aging' OR 'post-menopausal' OR 'postmenopaus*') OR 'sarcopenia' OR 'hand strength' OR 'weight' OR 'walking speed' OR 'muscle strength' OR 'physical activity').

AND

- Intervention: Omega-3 polyunsaturated fatty acid

('Eicosapentaenoic Acid' OR 'Docosahexaenoic Acid' OR 'Fatty Acids, Omega-3' OR 'Fatty Acids, Unsaturated' OR 'omega-3 fatty acid*' OR 'polyunsaturated fatty acid*' OR 'EPA' OR 'DHA' OR 'PUFA' OR 'omega-3').

AND

- Methodology: Randomised Control Trials

('randomised controlled trial' OR 'controlled clinical trial' OR 'randomised' OR 'placebo' OR 'clinical trials as topic' OR 'randomly' OR 'trial')

AND

- Humans: NOT 'animals/NOT humans'
