# Supplementary material for: Effect of dietary omega-3 fatty acid supplementation on frailty-related phenotypes in older adults: a systematic review and meta-analysis protocol
Source: BMJ Open. 2018 May 17;8(5):e021344. doi: 10.1136/bmjopen-2017-021344 (PMC5961576; doi:10.1136/bmjopen-2017-021344)
Supplement: Supplementary file 2 [file bmjopen-2017-021344supp002.pdf]

1	aged.mp. or Aged/
2	old.mp.
3	age-old.mp.
4	elder.mp.
5	senior.mp.
6	Functionally-Impaired.mp.
7	frail.mp.
8	exp Frail Elderly/
9	ageing.mp.
10	Aging/ or aging.mp.
11	post-menopausal.mp.
12	postmenopaus*.mp.
13	sarcopenia.mp. or Sarcopenia/
14	hand strength/ or handgrip strength.mp.
15	weight.mp.
16	walking speed.mp. or Walking Speed/
17	muscle strength.mp. or Muscle Strength/
18	physical activity.mp. or Exercise/
19	1 or 2 or 3 or 4 or 5 or 6 or 7 or 8 or 9 or 10 or 11 or 12 or 13 or 14 or 15 or 16 or 17 or 18
20	Eicosapentaenoic Acid.mp. or Eicosapentaenoic Acid/
21	Docosahexaenoic Acid.mp. or Docosahexaenoic Acids/
22	Fatty Acids, Omega-3.mp. or Fatty Acids, Omega-3/
23	Fatty Acids, Unsaturated.mp. or Fatty Acids, Unsaturated/
24	omega-3 fatty acid*.mp.
25	polyunsaturated fatty acid*.mp.
26	EPA.mp.
27	DHA.mp.
28	PUFA.mp.
29	omega-3.mp.
30	20 or 21 or 22 or 23 or 24 or 25 or 26 or 27 or 28 or 29
31	Randomi?ed controlled trial.mp.
32	Controlled clinical trial.mp
33	Randomised.mp
34	Placebo.mp.
35	Phase 4 clinical trial.mp.
36	Single Blind Procedure.mp.
37	Double Blind Procedure.mp.
38	Crossover Procedure.mp.
39	Clinical trials.mp.
40	Randomly.mp.
41	Trial.mp.
42	31 or 32 or 33 or 34 or 35 or 36 or 37 or 38 or 39 or 40 or 41 or 42
43	19 and 30 and 42
44	exp animals/ not humans.sh.
45	43 not 44

Box 1. Example of an advanced search strategy—MEDLINE OvidSP 1946 to 30th November 2017

Stages and detail of search strategy
