# Supplementary material for: Effect of dietary omega-3 fatty acid supplementation on frailty-related phenotypes in older adults: a systematic review and meta-analysis protocol
Source: BMJ Open. 2018 May 17;8(5):e021344. doi: 10.1136/bmjopen-2017-021344 (PMC5961576; doi:10.1136/bmjopen-2017-021344)
Supplement: Author's manuscript [file bmjopen-2017-021344.draft_revisions.pdf]

BMJ Open

BMJ Open is committed to open peer review. As part of this commitment we make the peer review history of every article we publish publicly available.

When an article is published we post the peer reviewers' comments and the authors' responses online. We also post the versions of the paper that were used during peer review. These are the versions that the peer review comments apply to.

The versions of the paper that follow are the versions that were submitted during the peer review process. They are not the versions of record or the final published versions. They should not be cited or distributed as the published version of this manuscript.

BMJ Open is an open access journal and the full, final, typeset and author-corrected version of record of the manuscript is available on our site with no access controls, subscription charges or pay-per-view fees (http://bmjopen.bmj.com).

If you have any questions on BMJ Open's open peer review process please email info.bmjopen@bmj.com

BMJ Open

Effect of dietary omega-3 fatty acid supplementation on frailty related phenotypes in older adults: a systematic review and meta-analysis protocol.

Journal:	*BMJ Open*
Manuscript ID	bmjopen-2017-021344
Article Type:	Protocol
Date Submitted by the Author:	21-Dec-2017
Complete List of Authors:	Stocks, Joanne; University of Nottingham, Academic Rheumatology<br>Valdes, A; University of Nottingham,
Keywords:	Frailty, Omega-3, Older people

SCHOLARONE™
Manuscripts

For peer review only

EFFECT OF DIETARY OMEGA-3 FATTY ACID SUPPLEMENTATION ON FRAILTY RELATED PHENOTYPES IN OLDER ADULTS: A SYSTEMATIC REVIEW AND META-ANALYSIS PROTOCOL

***Joanne Stocks[123], Ana M Valdes[123]**

Author Affiliations

[1]NIHR Nottingham BRC, UK

[2]Arthritis Research UK Pain Centre, University of Nottingham, UK

[3]Division of Rheumatology, Orthopaedics and Dermatology, School of Medicine, University of Nottingham, UK

***Corresponding author:**

Joanne Stocks,

Arthritis Research UK Pain Centre,

Academic Rheumatology,

University of Nottingham,

Clinical Sciences Building,

City Hospital,

Nottingham.

NG5 1PB

Email: Joanne.Stocks@nottingham.ac.uk

Tel: 0115 8231758

Word Count: 2034

Key Words: Frailty, Omega-3, Older adults

For peer review only

ABSTRACT

Introduction

The beneficial effect of dietary omega-3 supplementation in younger adults, or older people with acute or chronic disease is established. Knowledge is now needed about the effect in medically stable older people. The objective of this study is to examine and assess the evidence for a role of dietary omega-3 polyunsaturated fatty acid (PUFA) supplementation in older adults on (1) muscle mass and muscle strength (2) inflammatory biomarkers and (3) physical activity.

Methods and analysis

A systematic review and data synthesis will be conducted of randomised controlled trials in medically stable older people. Placebo controlled studies reporting interventions involving dietary supplementation of omega-3 PUFAs, eicosapentaenoic acid (EPA) and docosahexaenoic acid (DHA) will be included. Outcomes must include changes from baseline to last available follow-up for one or more of the following: muscle mass; inflammatory biomarkers; physical activity; walking speed; hand grip strength or muscle strength. Once the search strategy has been carried out, two independent researchers will assess relevant papers for eligibility. Articles up until 31st December 2017 in any language will be included. We will provide a narrative synthesis of the findings from the included studies. Studies will be grouped for meta-analysis according to the outcome(s) provided. Where studies have used the same type of intervention, with the same outcome measure, we will pool the results using a random-effects meta-analysis, with standardised mean differences for continuous outcomes and risk ratios for binary outcomes, and calculate 95% confidence intervals and two-sided P values for each outcome.

Ethics and dissemination

No research ethics approval is required for this systematic review as no confidential patient data will be used. The results of this systematic review will be disseminated through publication in an open access peer-reviewed journal and through conference presentations.

PROSPERO registration number: CRD42017080240

For peer review only

STRENGTHS AND LIMITATIONS OF THIS STUDY

- This will be the first study to systematically examine the effect of dietary omega-3 fatty acid supplementation on frailty related phenotypes in healthy older adults.
- Results will help to inform future guidelines on dietary supplementation for older adults.
- Limitations may include issues of poor reporting affecting risk of bias assessment and confidence in results.

INTRODUCTION

According to the United Nations, the number of people aged over 60 will double globally from 962 million in 2017 to 2.1 billion in 2050.[1] In Europe, the proportion of the population aged over 60 is projected to reach 35% by 2050.[1] It is, therefore, a global priority to ensure that this ageing population remains independent. A key element of maintaining independence in older adults is the preservation of mobility along with muscle mass and strength.

Muscle mass decline is one of the hallmarks of ageing and, from age 40 muscle mass begins to decrease, with an annual decline in functional capacity of up to 3% per year after age 60.[2] A key gerontological concept linked to musculoskeletal ageing is frailty.[3] The commonly acknowledged characteristics include unintentional weight loss, self-reported exhaustion, weakness (grip strength), slow walking speed, and low physical activity.[4] This complex phenomenon is highly correlated with loss of mobility along with progressive loss of skeletal muscle strength (dynapenia), mass and function (sarcopenia)[5] and results in a reduced quality of life and is a major public health concern.[6]

The prevalence of frailty also increases with age, and along with sarcopenia is associated with serious adverse outcomes, including falls, hospitalisation and mortality.[7] There is consensus of an inflammatory contribution to frailty. Striking differences in the levels of pro-inflammatory cytokines between frail and non-frail elderly have been reported,[8] and predict higher mortality.[9]

For peer review only

A role for nutritional determinants of frailty has been proposed[10] and a number of lifestyle interventions have been investigated with regards to frailty, including exercise and increased protein intake.[11]

Recent work has also begun to investigate such interventions to prevent or diminish muscle loss in medical settings, including the supplementation of leucine[12], vitamin D[13] and fish-derived polyunsaturated omega 3 fatty acids (PUFA), eicosapentaenoic acid (EPA) and docosahexaenoic acid (DHA). Studies carried out in a variety of populations including cancer patients,[14] patients with end-stage renal disease,[15] chronic obstructive pulmonary disease[16] and rheumatoid arthritis[17] have shown that dietary PUFAs have a beneficial effect on skeletal muscle mass and strength.

Dietary supplementation of omega-3 PUFAs is of particular interest in the context of frailty, given its well-known anti-inflammatory role and the importance of inflammation in the development of ageing.[18]

Omega-3 reduces inflammation in conditions including Duchenne muscular dystrophy,[19] Crohn's disease,[20] non-alcoholic fatty liver disease,[21] cardiovascular disease[22] as well as many cancers.[14 23-25] These studies are of particular interest, as increased levels of inflammatory biomarkers such as interleukin-6 (IL-6), tumour necrosis factor-alpha (TNFα) and C reactive protein (CRP) have all been linked with both frailty and sarcopenia in older adults.[26-28] A more pro-inflammatory diet has also been linked with a higher incidence of frailty.[29]

Whilst a recent review by Ticinesi *et al.*[18] reviewed the analysis of omega-3 PUFAs on inflammation in older individuals in both cross-sectional and randomized controlled trials, we are not aware of any systematic reviews or meta-analysis focussing specifically on omega-3 fatty acid supplementation on frailty phenotypes in older healthy adults. We propose to conduct a systematic review and meta-analysis to examine the effect of dietary omega-3 supplementation in medically stable older people. The objective of this systematic review is to examine and assess the evidence for a role of dietary omega-3 fatty acid

For peer review only

supplementation in older adults on (1) muscle mass and muscle strength (2) inflammatory biomarkers and (3) physical activity, as well as biases in the included studies.

METHODS AND ANALYSIS

Registration

This protocol has been registered with PROSPERO (registration number CRD42017080240[30]) and reported in accordance with Preferred Reporting Items for Systematic Reviews and Meta-Analyses (PRISMA)[31] and PRISMA-Protocol [32 33] guidelines.

Study selection criteria

Interventions and population

Studies reporting results of interventions involving dietary supplementation of omega-3 PUFAs will be included. Dietary supplementation will be defined as daily ingestion of capsules containing EPA and DHA or through an EPA and DHA enriched diet. The comparator will be placebo-controlled groups. Participants will be medically stable and include community-dwelling, hospitalised or institutionalised persons, of either sex, classified by the study authors as postmenopausal or older people with the majority of participants over 60 years of age. To ensure the focus of the review is on medically stable healthy older people, exclusions will be studies where participants were selected because they had a cancer or other chronic disease diagnosis. Participants who currently consumed a high fish diet or use fish oil supplements will also be excluded.

Outcomes

Outcomes must include changes from baseline to last available follow-up for one or more of the following: muscle mass; inflammatory biomarkers; physical activity; walking speed; hand grip strength or muscle strength. Any adverse effects will also be summarised. Studies will be grouped for meta-analysis according to the outcome(s) provided.

Study designs

Randomised controlled trials (RCTs) will be included.

For peer review only

Other

Articles up until 31st December 2017 in any language will be included.

Exclusion criteria

Studies will be excluded for the following reasons: (1) study population was focused on participants diagnosed and being treated for a pre-existing medical condition (e.g. cancer, kidney disease, liver disease, diabetes mellitus, cardiovascular disease); or (2) letters to the editor, meta-analyses, case reports and reviews.

Search strategy

MEDLINE (OVID) from 1946, the Cochrane Register of Controlled Trials (CENTRAL) from 1940, EMBASE from 1946, Cumulative Index to Nursing and Allied Health Literature (CINAHL) from 1937, Allied and Complementary Medicine Database (AMED) and Web of Science will be searched for relevant trials. The search strategy for Medline has been developed in consultation with a subject-specific librarian and will be adapted for use in other databases. Search terms are informed by Cochrane Handbook[34] and other systematic reviews investigating PUFA dietary supplementation, sarcopenia and/or frailty.

Study population terms:

- Population and target condition: Older people and frailty

('aged' OR 'old' OR 'age-old' OR 'elder' OR 'senior' OR 'functionally impaired' OR 'frail' OR 'exp frail elderly' OR 'ageing' OR 'aging' OR 'post-menopausal' OR 'postmenopaus*') OR 'sarcopenia').

AND

- Intervention: Omega-3 polyunsaturated fatty acid

('Eicosapentaenoic Acid' OR 'Docosahexaenoic Acid' OR 'Fatty Acids, Omega-3' OR 'Fatty Acids, Unsaturated' OR 'omega-3 fatty acid*' OR 'polyunsaturated fatty acid*' OR 'EPA' OR 'DHA' OR 'PUFA' OR 'omega-3').

AND

- Methodology: Randomised Control Trials

('randomised controlled trial' OR 'controlled clinical trial' OR 'randomised' OR 'placebo' OR 'clinical trials as topic' OR 'randomly' OR 'trial')

For peer review only

AND

- Humans: NOT 'animals/NOT humans'

Example of searches that will be used can be seen in Box 1 MEDLINE (OVID) advanced search. Syntax (truncation, wildcards and quotation marks) and operators will be amended according to the specific databases. Initial search results will be uploaded to EndNote X7 (Thomas Reuters) prior to the review of titles and abstracts.

Data extraction

Initial title and abstract review will be conducted by the first author (JS). Duplicates and articles clearly not meeting the selection criteria will be removed. The reference lists from identified letters to the editor, meta-analyses, case reports and reviews will be scanned to identify further trials. Two-independent researchers (JS and AMV) will then read the full text of remaining relevant papers for eligibility. In cases where the two researchers cannot agree on eligibility, a third researcher will mediate. Authors of grey literature will be contacted when conference abstracts and proceedings are found. A PRISMA flowchart will be used to provide transparency of the number of papers included or excluded at each stage. Two-independent researchers (JS and AMV) will extract the data. The data extracted from the studies (if available) will include (1) authors; (2) publication year; (3) country; (4) funding; (5) setting; (6) study design; (7) sample size; (8) duration of monitoring or intervention; (9) withdrawals; (10) mean age; (11) gender; (12) muscle mass; (13) physical activity; (14) muscle strength; (15) walking speed; (16) handgrip strength and (17) biomarkers;

Risk of bias assessment

Reporting bias will be assessed by plotting the inverse of the SEs of the effect estimates using funnel plots where meta-analysis includes more than 10 trials and will be assessed visually for asymmetry[35] and with the Egger's regression test for continuous variables.[36] Analysis will be conducted on Review Manager Software.[37]

JS and AMV will independently assess the risk of study bias using the Cochrane Collaboration's tool for assessing risk of bias in randomised trials.[38] The Cochrane risk of bias tool for RCT's consists of the following seven items: (1) random sequence generation; (2)

For peer review only

allocation concealment; (3) blinding of participants and personnel; (4) blinding of outcome assessment; (5) incomplete outcome data; (6) selective reporting; (7) other sources of bias. Questions are rated as having a high, low or unclear level of bias across the seven domains.

Data management and statistical analysis

Data obtained through data extraction will be entered into Excel. Outcomes will be imported into RevMan[37] for meta-analysis. Data extracted must be presented as mean and standard deviation, not ranges, and will not be estimated from graphs or figures. Authors will be contacted if mean and standard deviation values are not presented.

We will create a table describing study characteristics and major outcomes. We will provide a narrative synthesis of the findings from the included studies, structured around the type and content of intervention (i.e. diet alone or in combination with training), target population characteristics (i.e. sex, age, body mass index (BMI)), type of outcome (i.e. muscle strength; physical performance; muscle mass; cognitive function). We will provide summaries of intervention effects for each study by calculating risk ratios (for dichotomous outcomes) or standardised mean differences (for continuous outcomes).

We anticipate that there will be limited scope for meta-analysis because of the range of different outcomes measured across the small number of existing trials. However, where studies have used the same type of intervention, with the same outcome measure, we will pool the results using a random-effects meta-analysis, with standardised mean differences for continuous outcomes and risk ratios for binary outcomes, and calculate 95% confidence intervals and two-sided P values for each outcome. In studies where the effects of clustering have not been taken into account, we will adjust the standard deviations for the design effect. Heterogeneity between the studies in effect measures will be assessed using the I^2 statistic.[39] We will consider an I^2 value greater than 50% indicative of moderate heterogeneity or 75% high heterogeneity.[40]

We will conduct sensitivity analyses based on study quality. We will use stratified meta-analyses to explore heterogeneity in effect estimates according to participant characteristics

(e.g. sex, age, BMI), location (e.g. hospital or community setting), intervention components (e.g. diet alone or in combination with training), the logistics of intervention provision.

Outcomes and prioritisation.

The primary outcomes will include recognised frailty criteria of physical activity levels; walking speed; hand-grip strength or muscle strength; and weight[4] along with changes in muscle mass and circulating levels of the pro-inflammatory markers CRP, IL-6 and TNF-α. Other outcomes will be analysed if available including body fat mass.

ETHICS AND DISSEMINATION

No research ethics approval is required for this systematic review as no confidential patient data will be used. It is intended that the results of this systematic review will be disseminated through publication in an open access peer-reviewed journal and through conference presentations. All amendments to the protocol will be documented and dated and reported in the PROSPERO trial registry.

DISCUSSION

This systematic review will utilise rigorous methodology, to identify and examine studies reporting the outcome of omega-3 supplementation on frailty related traits on healthy ageing groups, including both inflammatory biomarkers and functional measures. No systematic review has previously addressed this objective although numerous published reviews have focused on ageing populations suffering from chronic or acute conditions. For example, there is evidence of beneficial effects of omega-3 supplementation for individuals undergoing chemotherapy or radiotherapy for cancer[41] for risk reduction in individuals with established atherosclerotic cardiovascular disease[42] and some beneficial effect on liver function in individuals with non-alcoholic fatty liver disease[43] among others.

Although risk of bias and overall level of evidence may limit analyses and confidence in this review's conclusions, this synthesis will provide a better understanding of the effect of omega-3 supplementation in preventing systemic inflammation and functional decline in the healthy elderly population.

Implications of results

This review will provide the first rigorous summary of effect of omega-3 supplementation across all published randomized controlled trial studies of healthy elderly individuals. The findings will inform our understanding of the value of this popular nutritional supplement in preventing frailty-related outcomes.

REFERENCES

1. United Nations D. World Population Prospects: The 2017 Revision, Key Findings and Advance Tables. Department of Economic and Social Affairs, Population Division 2017.
2. Patel HP, Syddall HE, Jameson K, et al. Prevalence of sarcopenia in community-dwelling older people in the UK using the European Working Group on Sarcopenia in Older People (EWGSOP) definition: findings from the Hertfordshire Cohort Study (HCS). *Age Ageing* 2013;42(3):378-84. doi: 10.1093/ageing/afs197
3. Davies B, Garcia F, Ara I, et al. Relationship Between Sarcopenia and Frailty in the Toledo Study of Healthy Aging: A Population Based Cross-Sectional Study. *J Am Med Dir Assoc* 2017 doi: 10.1016/j.jamda.2017.09.014
4. Fried LP, Young Y, Rubin G, et al. Self-reported preclinical disability identifies older women with early declines in performance and early disease. *J Clin Epidemiol* 2001;54(9):889-901.
5. Morley JE, Baumgartner RN, Roubenoff R, et al. Sarcopenia. *J Lab Clin Med* 2001;137(4):231-43. doi: 10.1067/mlc.2001.113504
6. Cruz-Jentoft AJ, Baeyens JP, Bauer JM, et al. Sarcopenia: European consensus on definition and diagnosis: Report of the European Working Group on Sarcopenia in Older People. *Age Ageing* 2010;39(4):412-23. doi: 10.1093/ageing/afq034
7. Balogun S, Winzenberg T, Wills K, et al. Prospective Associations of Low Muscle Mass and Function with 10-Year Falls Risk, Incident Fracture and Mortality in Community-Dwelling Older Adults. *J Nutr Health Aging* 2017;21(7):843-48. doi: 10.1007/s12603-016-0843-6
8. Leng SX, Xue QL, Tian J, et al. Inflammation and frailty in older women. *J Am Geriatr Soc* 2007;55(6):864-71. doi: 10.1111/j.1532-5415.2007.01186.x
9. Soysal P, Stubbs B, Lucato P, et al. Inflammation and frailty in the elderly: A systematic review and meta-analysis. *Ageing Res Rev* 2016;31:1-8. doi: 10.1016/j.arr.2016.08.006
10. Lorenzo-Lopez L, Maseda A, de Labra C, et al. Nutritional determinants of frailty in older adults: A systematic review. *BMC Geriatr* 2017;17(1):108. doi: 10.1186/s12877-017-0496-2
11. Puts MTE, Toubasi S, Andrew MK, et al. Interventions to prevent or reduce the level of frailty in community-dwelling older adults: a scoping review of the literature and international policies. *Age Ageing* 2017;46(3):383-92. doi: 10.1093/ageing/afw247

For peer review only

12. Wilkinson DJ, Bukhari SSI, Phillips BE, et al. Effects of leucine-enriched essential amino acid and whey protein bolus dosing upon skeletal muscle protein synthesis at rest and after exercise in older women. *Clin Nutr* 2017 doi: 10.1016/j.clnu.2017.09.008
13. Englund DA, Kirn DR, Koochek A, et al. Nutritional Supplementation With Physical Activity Improves Muscle Composition in Mobility-Limited Older Adults, The VIVE2 Study: A Randomized, Double-Blind, Placebo-Controlled Trial. *J Gerontol A Biol Sci Med Sci* 2017 doi: 10.1093/gerona/glx141
14. Pappalardo G, Almeida A, Ravasco P. Eicosapentaenoic acid in cancer improves body composition and modulates metabolism. *Nutrition* 2015;31(4):549-55. doi: 10.1016/j.nut.2014.12.002
15. Wong TC, Chen YT, Wu PY, et al. Ratio of Dietary n-6/n-3 Polyunsaturated Fatty Acids Independently Related to Muscle Mass Decline in Hemodialysis Patients. *PLoS One* 2015;10(10):e0140402. doi: 10.1371/journal.pone.0140402
16. van de Bool C, Rutten EPA, van Helvoort A, et al. A randomized clinical trial investigating the efficacy of targeted nutrition as adjunct to exercise training in COPD. *J Cachexia Sarcopenia Muscle* 2017;8(5):748-58. doi: 10.1002/jcsm.12219
17. Berbert AA, Kondo CR, Almendra CL, et al. Supplementation of fish oil and olive oil in patients with rheumatoid arthritis. *Nutrition* 2005;21(2):131-6. doi: 10.1016/j.nut.2004.03.023
18. Ticinesi A, Meschi T, Lauretani F, et al. Nutrition and Inflammation in Older Individuals: Focus on Vitamin D, n-3 Polyunsaturated Fatty Acids and Whey Proteins. *Nutrients* 2016;8(4):186. doi: 10.3390/nu8040186
19. Rodriguez-Cruz M, Cruz-Guzman ODR, Almeida-Becerril T, et al. Potential therapeutic impact of omega-3 long chain-polyunsaturated fatty acids on inflammation markers in Duchenne muscular dystrophy: A double-blind, controlled randomized trial. *Clin Nutr* 2017 doi: 10.1016/j.clnu.2017.09.011
20. Yao J, Lu Y, Zhi M, et al. Dietary n3 polyunsaturated fatty acids ameliorate Crohn's disease in rats by modulating the expression of PPARgamma/NFAT. *Molecular medicine reports* 2017 doi: 10.3892/mmr.2017.7673 [published Online First: 2017/10/11]
21. Wang C, Liu W, Yao L, et al. Hydroxyeicosapentaenoic acids and epoxyeicosatetraenoic acids attenuate early occurrence of nonalcoholic fatty liver disease. *Br J Pharmacol* 2017;174(14):2358-72. doi: 10.1111/bph.13844
22. Siddiqui RA, Harvey KA, Ruzmetov N, et al. n-3 fatty acids prevent whereas trans-fatty acids induce vascular inflammation and sudden cardiac death. *Br J Nutr* 2009;102(12):1811-9. doi: 10.1017/S0007114509992030
23. Chung H, Lee YS, Mayoral R, et al. Omega-3 fatty acids reduce obesity-induced tumor progression independent of GPR120 in a mouse model of postmenopausal breast cancer. *Oncogene* 2015;34(27):3504-13. doi: 10.1038/onc.2014.283
24. Miccadei S, Masella R, Mileo AM, et al. omega3 Polyunsaturated Fatty Acids as Immunomodulators in Colorectal Cancer: New Potential Role in Adjuvant Therapies. *Front Immunol* 2016;7:486. doi: 10.3389/fimmu.2016.00486
25. Finocchiaro C, Segre O, Fadda M, et al. Effect of n-3 fatty acids on patients with advanced lung cancer: a double-blind, placebo-controlled study. *Br J Nutr* 2012;108(2):327-33. doi: 10.1017/S0007114511005551

For peer review only

26. Coto Montes A, Boga JA, Bermejo Millo C, et al. Potential early biomarkers of sarcopenia among independent older adults. *Maturitas* 2017;104:117-22. doi: 10.1016/j.maturitas.2017.08.009
27. Hsu B, Hirani V, Cumming RG, et al. Cross-Sectional and Longitudinal Relationships Between Inflammatory Biomarkers and Frailty in Community-dwelling Older Men: The Concord Health and Ageing in Men Project. *J Gerontol A Biol Sci Med Sci* 2017 doi: 10.1093/gerona/glx142
28. Hida T, Imagama S, Ando K, et al. Sarcopenia and physical function are associated with inflammation and arteriosclerosis in community-dwelling people: The Yakumo study. *Mod Rheumatol* 2017:1-6. doi: 10.1080/14397595.2017.1349058
29. Shivappa N, Stubbs B, Hebert JR, et al. The Relationship Between the Dietary Inflammatory Index and Incident Frailty: A Longitudinal Cohort Study. *J Am Med Dir Assoc* 2017 doi: 10.1016/j.jamda.2017.08.006
30. Stocks J, Valdes AM. Effect of dietary omega-3 fatty acid supplementation on frailty related phenotypes in older adults: a systematic review and meta-analysis. PROSPERO CRD42017080240. 2017.
31. Moher D, Liberati A, Tetzlaff J, et al. Preferred reporting items for systematic reviews and meta-analyses: the PRISMA statement. *J Clin Epidemiol* 2009;62(10):1006-12. doi: 10.1016/j.jclinepi.2009.06.005
32. Moher D, Shamseer L, Clarke M, et al. Preferred reporting items for systematic review and meta-analysis protocols (PRISMA-P) 2015 statement. *Syst Rev* 2015;4:1. doi: 10.1186/2046-4053-4-1
33. Shamseer L, Moher D, Clarke M, et al. Preferred reporting items for systematic review and meta-analysis protocols (PRISMA-P) 2015: elaboration and explanation. *BMJ* 2015;349:g7647. doi: 10.1136/bmj.g7647
34. Lefebvre C ME, Glanville J. Searching for studies. In: Higgins J GS, ed. Cochrane Handbook for Systematic Reviews of Interventions Version 510 The Cochrane Collaboration, 2011.
35. Sutton AJ, Duval SJ, Tweedie RL, et al. Empirical assessment of effect of publication bias on meta-analyses. *Brit Med J* 2000;320(7249):1574-77. doi: DOI 10.1136/bmj.320.7249.1574
36. Egger M, Davey Smith G, Schneider M, et al. Bias in meta-analysis detected by a simple, graphical test. *BMJ* 1997;315(7109):629-34.
37. Review Manager (RevMan) [program]. Version 5.3 version. Copenhagen: The Nordic Cochrane Centre, : The Cochrane Collaboration, 2014.
38. Higgins JP, Altman DG, Gotzsche PC, et al. The Cochrane Collaboration's tool for assessing risk of bias in randomised trials. *BMJ* 2011;343:d5928. doi: 10.1136/bmj.d5928
39. Higgins JP, Thompson SG. Quantifying heterogeneity in a meta-analysis. *Stat Med* 2002;21(11):1539-58. doi: 10.1002/sim.1186
40. Higgins JP, Thompson SG, Spiegelhalter DJ. A re-evaluation of random-effects meta-analysis. *J R Stat Soc Ser A Stat Soc* 2009;172(1):137-59. doi: 10.1111/j.1467-985X.2008.00552.x
41. de Aguiar Pastore Silva J, Emilia de Souza Fabre M, Waitzberg DL. Omega-3 supplements for patients in chemotherapy and/or radiotherapy: A systematic review. *Clin Nutr* 2015;34(3):359-66. doi: 10.1016/j.clnu.2014.11.005

For peer review only

42. Burke MF, Burke FM, Soffer DE. Review of Cardiometabolic Effects of Prescription Omega-3 Fatty Acids. *Curr Atheroscler Rep* 2017;19(12):60. doi: 10.1007/s11883-017-0700-z
43. Yu L, Yuan M, Wang L. The effect of omega-3 unsaturated fatty acids on non-alcoholic fatty liver disease: A systematic review and meta-analysis of RCTs. *Pak J Med Sci* 2017;33(4):1022-28. doi: 10.12669/pjms.334.12315

Authors' contributions: JS, the guarantor of the protocol, drafted the protocol and registered it in PROSPERO. JS and AV drafted the manuscript, contributed to the development of the selection criteria, the risk of bias assessment strategy, data extraction criteria and search strategy. All authors read, provided feedback and approved the final manuscript.

Acknowledgement: The authors thank Professor Jo Leonardi-Bee for her critical review of the manuscript.

Funding statement: No funding has been received for this study.

Competing interests statement: None declared

For peer review only

1	aged.mp. or Aged/
2	old.mp.
3	age-old.mp.
4	elder.mp.
5	senior.mp.
6	Functionally-Impaired.mp.
7	frail.mp.
8	exp Frail Elderly/
9	ageing.mp.
10	Aging/ or aging.mp.
11	post-menopausal.mp.
12	postmenopaus*.mp.
13	sarcopenia.mp. or Sarcopenia/
14	1 or 2 or 3 or 4 or 5 or 6 or 7 or 8 or 9 or 10 or 11 or 12 or 13
15	Eicosapentaenoic Acid.mp. or Eicosapentaenoic Acid/
16	Docosahexaenoic Acid.mp. or Docosahexaenoic Acids/
17	Fatty Acids, Omega-3.mp. or Fatty Acids, Omega-3/
18	Fatty Acids, Unsaturated.mp. or Fatty Acids, Unsaturated/
19	omega-3 fatty acid*.mp.
20	polyunsaturated fatty acid*.mp.
21	EPA.mp.
22	DHA.mp.
23	PUFA.mp.
24	omega-3.mp.
25	15 or 16 or 17 or 18 or 19 or 20 or 21 or 22 or 23 or 24
26	Randomi?ed controlled trial.mp.
27	Controlled clinical trial.mp
28	Randomised.mp
29	Placebo.mp.
30	Phase 4 clinical trial.mp.
31	Single Blind Procedure.mp.
32	Double Blind Procedure.mp.
33	Crossover Procedure.mp.
34	Placebo.mp.
35	Clinical trials.mp.
36	Randomly.mp.
37	Trial.mp.
39	26 or 27 or 28 or 29 or 30 or 31 or 32 or 33 or 34 or 35 or 36 or 37
40	14 and 25 and 39
41	exp animals/ not humans.sh.
42	40 not 41

Box 1. Example of an advanced search strategy—MEDLINE OvidSP 1946 to 30th November 2017

Stages and detail of search strategy

For peer review only

PRISMA-P 2015 Checklist

Section/topic	#	Checklist item	Information reported		Line number(s)
			Yes	No	
**ADMINISTRATIVE INFORMATION**					
**Title**					
Identification	1a	Identify the report as a protocol of a systematic review	X		P1, 3-5
Update	1b	If the protocol is for an update of a previous systematic review, identify as such			
**Registration**	2	If registered, provide the name of the registry (e.g., PROSPERO) and registration number in the Abstract	X		P2, 49
**Authors**					
Contact	3a	Provide name, institutional affiliation, and e-mail address of all protocol authors; provide physical mailing address of corresponding author	X		P1, 8-39
Contributions	3b	Describe contributions of protocol authors and identify the guarantor of the review	X		P12, 33-40
**Amendments**	4	If the protocol represents an amendment of a previously completed or published protocol, identify as such and list changes; otherwise, state plan for documenting important protocol amendments			
**Support**					
Sources	5a	Indicate sources of financial or other support for the review	X		P12, 49
Sponsor	5b	Provide name for the review funder and/or sponsor	X		P12, 49
Role of sponsor/funder	5c	Describe roles of funder(s), sponsor(s), and/or institution(s), if any, in developing the protocol		X	
**INTRODUCTION**					
**Rationale**	6	Describe the rationale for the review in the context of what is already known	X		P3-4, 21-53
**Objectives**	7	Provide an explicit statement of the question(s) the review will address with reference to participants, interventions, comparators, and outcomes (PICO)	X		P2, 10-15
**METHODS**					
**Eligibility criteria**	8	Specify the study characteristics (e.g., PICO, study design, setting, time frame) and report	X		P5-6, 18-15

BioMed Central The Open Access Publisher

For peer review only

Section/topic	#	Checklist item	Information reported Yes	Information reported No	Line number(s)
		characteristics (e.g., years considered, language, publication status) to be used as criteria for eligibility for the review			
**Information sources**	9	Describe all intended information sources (e.g., electronic databases, contact with study authors, trial registers, or other grey literature sources) with planned dates of coverage	☒	☐	P6, 20-25
**Search strategy**	10	Present draft of search strategy to be used for at least one electronic database, including planned limits, such that it could be repeated	☒	☐	P13
STUDY RECORDS					
Data management	11a	Describe the mechanism(s) that will be used to manage records and data throughout the review	☒	☐	P7, 10-11<br>P8, 10-12
Selection process	11b	State the process that will be used for selecting studies (e.g., two independent reviewers) through each phase of the review (i.e., screening, eligibility, and inclusion in meta-analysis)	☒	☐	P7, 17-29
Data collection process	11c	Describe planned method of extracting data from reports (e.g., piloting forms, done independently, in duplicate), any processes for obtaining and confirming data from investigators	☒	☐	P7, 29-31<br>P8, 11-15
**Data items**	12	List and define all variables for which data will be sought (e.g., PICO items, funding sources), any pre-planned data assumptions and simplifications	☒	☐	P7, 32-38
**Outcomes and prioritization**	13	List and define all outcomes for which data will be sought, including prioritization of main and additional outcomes, with rationale	☒	☐	P9, 6-12
**Risk of bias in individual studies**	14	Describe anticipated methods for assessing risk of bias of individual studies, including whether this will be done at the outcome or study level, or both; state how this information will be used in data synthesis	☒	☐	P7-8, 42-5
DATA					
**Synthesis**	15a	Describe criteria under which study data will be quantitatively synthesized	☒	☐	P8 34-38
	15b	If data are appropriate for quantitative synthesis, describe planned summary measures, methods of handling data, and methods of combining data from studies, including any planned exploration of consistency (e.g., I^2, Kendall's tau)	☒	☐	P8, 36-50
	15c	Describe any proposed additional analyses (e.g., sensitivity or subgroup analyses, meta-regression)	☒	☐	P8-9, 53-5
	15d	If quantitative synthesis is not appropriate, describe the type of summary planned	☒	☐	P8, 20-31

BioMed Central
The Open Access Publisher

For peer review only

Section/topic	#	Checklist item	Information reported		Line number(s)
			Yes	No	
**Meta-bias(es)**	16	Specify any planned assessment of meta-bias(es) (e.g., publication bias across studies, selective reporting within studies)	☒	☐	P7-8, 43-6
**Confidence in cumulative evidence**	17	Describe how the strength of the body of evidence will be assessed (e.g., GRADE)	☐	☐	

For peer review only

BioMed Central
The Open Access Publisher

BMJ Open

Effect of dietary omega-3 fatty acid supplementation on frailty related phenotypes in older adults: a systematic review and meta-analysis protocol.

Journal:	*BMJ Open*
Manuscript ID	bmjopen-2017-021344.R1
Article Type:	Protocol
Date Submitted by the Author:	21-Feb-2018
Complete List of Authors:	Stocks, Joanne; University of Nottingham, Academic Rheumatology<br>Valdes, A; University of Nottingham,
**Primary Subject Heading**:	Geriatric medicine
Secondary Subject Heading:	Epidemiology, Nutrition and metabolism
Keywords:	Frailty, Omega-3, Older people

SCHOLARONE™ Manuscripts

EFFECT OF DIETARY OMEGA-3 FATTY ACID SUPPLEMENTATION ON FRAILTY RELATED PHENOTYPES IN OLDER ADULTS: A SYSTEMATIC REVIEW AND META-ANALYSIS PROTOCOL

***Joanne Stocks[123], Ana M Valdes[123]**

Author Affiliations

[1]NIHR Nottingham BRC, UK

[2]Arthritis Research UK Pain Centre, University of Nottingham, UK

[3]Division of Rheumatology, Orthopaedics and Dermatology, School of Medicine, University of Nottingham, UK

***Corresponding author:**

Joanne Stocks,

Arthritis Research UK Pain Centre,

Academic Rheumatology,

University of Nottingham,

Clinical Sciences Building,

City Hospital,

Nottingham.

NG5 1PB

Email: Joanne.Stocks@nottingham.ac.uk

Tel: 0115 8231758

Word Count: 2186

Key Words: Frailty, Omega-3, Older adults

For peer review only

ABSTRACT

Introduction

The beneficial effect of dietary omega-3 supplementation in younger adults, or older people with acute or chronic disease is established. Knowledge is now needed about the effect in medically stable older people. The objective of this study is to examine and assess the evidence for a role of dietary omega-3 polyunsaturated fatty acid (PUFA) supplementation in older adults on (1) muscle mass and muscle strength (2) inflammatory biomarkers and (3) physical activity.

Methods and analysis

A systematic review and data synthesis will be conducted of randomised controlled trials in older people not recruited for any given disease diagnosis. Placebo controlled studies reporting interventions involving dietary supplementation of omega-3 PUFAs, eicosapentaenoic acid (EPA) and docosahexaenoic acid (DHA) will be included. Outcomes must include changes from baseline to last available follow-up for one or more of the following: muscle mass; inflammatory biomarkers; physical activity; walking speed; weight change; hand grip strength or muscle strength. Once the search strategy has been carried out, two independent researchers will assess relevant papers for eligibility. Articles up until 31st December 2017 in any language will be included. We will provide a narrative synthesis of the findings from the included studies. Studies will be grouped for meta-analysis according to the outcome(s) provided. Where studies have used the same type of intervention, with the same outcome measure, we will pool the results using a random-effects meta-analysis, with standardised mean differences for continuous outcomes and risk ratios for binary outcomes, and calculate 95% confidence intervals and two-sided P values for each outcome.

Ethics and dissemination

No research ethics approval is required for this systematic review, as no confidential patient data will be used. The results of this systematic review will be disseminated through publication in an open access peer-reviewed journal and through conference presentations.

PROSPERO registration number: CRD42017080240

For peer review only - http://bmjopen.bmj.com/site/about/guidelines.xhtml

STRENGTHS AND LIMITATIONS OF THIS STUDY

- This will be the first study to systematically examine the effect of dietary omega-3 fatty acid supplementation on frailty related phenotypes in healthy older adults.
- Results will help to inform future guidelines on dietary supplementation for older adults.
- Limitations may include issues of poor reporting affecting risk of bias assessment and confidence in results.

INTRODUCTION

According to the United Nations, the number of people aged over 60 will double globally from 962 million in 2017 to 2.1 billion in 2050.[1] In Europe, the proportion of the population aged over 60 is projected to reach 35% by 2050.[1] It is, therefore, a global priority to ensure that this ageing population remains independent. A key element of maintaining independence in older adults is the preservation of mobility along with muscle mass and strength.

Muscle mass decline is one of the hallmarks of ageing and, from age 40 muscle mass begins to decrease, with an annual decline in functional capacity of up to 3% per year after age 60.[2] A key gerontological concept linked to musculoskeletal ageing is frailty.[3] The commonly acknowledged characteristics include unintentional weight loss, self-reported exhaustion, weakness (grip strength), slow walking speed, and low physical activity.[4] This complex phenomenon is highly correlated with loss of mobility along with progressive loss of skeletal muscle strength (dynapenia), mass and function (sarcopenia)[5] and results in a reduced quality of life and is a major public health concern.[6]

The prevalence of frailty also increases with age, and along with sarcopenia is associated with serious adverse outcomes, including falls, hospitalisation and mortality.[7] There is consensus of an inflammatory contribution to frailty. Striking differences in the levels of pro-inflammatory cytokines between frail and non-frail elderly have been reported,[8] and predict higher mortality.[9]

For peer review only

A role for nutritional determinants of frailty has been proposed[10] and a number of lifestyle interventions have been investigated with regards to frailty, including exercise and increased protein intake.[11]

Recent work has also begun to investigate such interventions to prevent or diminish muscle loss in medical settings, including the supplementation of leucine[12], vitamin D[13] and fish-derived polyunsaturated omega 3 fatty acids (PUFA), eicosapentaenoic acid (EPA) and docosahexaenoic acid (DHA). Studies carried out in a variety of populations including cancer patients,[14] patients with end-stage renal disease,[15] chronic obstructive pulmonary disease[16] and rheumatoid arthritis[17] have shown that dietary PUFAs have a beneficial effect on skeletal muscle mass and strength.

Dietary supplementation of omega-3 PUFAs is of particular interest in the context of frailty, given its well-known anti-inflammatory role and the importance of inflammation in the development of ageing.[18]

Omega-3 reduces inflammation in conditions including Duchenne muscular dystrophy,[19] Crohn's disease,[20] non-alcoholic fatty liver disease,[21] cardiovascular disease[22] as well as many cancers.[14 23-25] These studies are of particular interest, as increased levels of inflammatory biomarkers such as interleukin-6 (IL-6), tumour necrosis factor-alpha (TNFα) and C reactive protein (CRP) have all been linked with both frailty and sarcopenia in older adults.[26-28] Long chain PUFAs have been suggested to interact with antioxidants and improve inflammatory responses to positively impact on physical performance.[29] Part of the mechanism may involve the effect of omega-3 on musculoskeletal pain; a pain reduction would be conducive to more physical activity. [30] More generally, omega-3 supplementation may act directly on skeletal muscle and improve protein metabolism hence having an influence on physical performance. [31 32] A more pro-inflammatory diet has also been linked with a higher incidence of frailty.[33]

A recent review by Ticinesi *et al.*[18] summarised the analysis of omega-3 PUFAs on inflammation in older individuals in both cross-sectional and randomized controlled trials. However, we are not aware of any systematic reviews or meta-analyses focussing

For peer review only

specifically on omega-3 fatty acid supplementation on frailty phenotypes in older healthy adults. We propose to conduct a systematic review and meta-analysis to examine the effect of dietary omega-3 supplementation in older people not recruited for any given disease diagnosis. The outcomes that will be investigated are inflammatory biomarkers; muscle mass; physical activity; walking speed; weight change; hand grip strength or muscle strength, as well as biases in the included studies.

METHODS AND ANALYSIS

Registration

This protocol has been registered with PROSPERO (registration number CRD42017080240[34]) and reported in accordance with Preferred Reporting Items for Systematic Reviews and Meta-Analyses (PRISMA)[35] and PRISMA-Protocol [36 37] guidelines.

Study selection criteria

Interventions and population

Studies reporting results of interventions involving dietary supplementation of omega-3 PUFAs will be included. Dietary supplementation will be defined as daily ingestion of capsules containing EPA and DHA or through an EPA and DHA enriched diet. The comparator will be placebo-controlled groups. Participants will include community-dwelling, hospitalised or institutionalised persons, of either sex, classified by the study authors as postmenopausal or older people with the majority of participants over 60 years of age. To ensure the focus of the review is on older people not recruited for any given disease diagnosis, exclusions will be studies where participants were selected because they had a cancer or other chronic disease diagnosis. Participants who currently consumed a high fish diet or use fish oil supplements will also be excluded.

Outcomes

Outcomes must include changes from baseline to last available follow-up for one or more of the following: muscle mass; inflammatory biomarkers; physical activity; walking speed; weight change; hand grip strength or muscle strength. Any adverse effects will also be summarised. Studies will be grouped for meta-analysis according to the outcome(s) provided.

For peer review only

Study designs

Randomised controlled trials (RCTs) will be included.

Other

Articles up until 31st December 2017 in any language will be included.

Exclusion criteria

Studies will be excluded for the following reasons: (1) study population was specifically focused on participants diagnosed and being treated for a pre-existing medical condition (e.g. cancer, kidney disease, liver disease, diabetes mellitus, cardiovascular disease); or (2) letters to the editor, meta-analyses, case reports and reviews.

Search strategy

MEDLINE (OVID) from 1946, the Cochrane Register of Controlled Trials (CENTRAL) from 1940, EMBASE from 1946, Cumulative Index to Nursing and Allied Health Literature (CINAHL) from 1937, Allied and Complementary Medicine Database (AMED) and Web of Science will be searched for relevant trials. The search strategy for Medline has been developed in consultation with a subject-specific librarian and will be adapted for use in other databases. Search terms are informed by Cochrane Handbook[38] and other systematic reviews investigating PUFA dietary supplementation, sarcopenia and/or frailty.

The full search strategy can be found in the supplementary file. Example of searches that will be used can be seen in Supplementary file Box 1 MEDLINE (OVID) Advanced Search Example. Syntax (truncation, wildcards and quotation marks) and operators will be amended according to the specific databases. Initial search results will be uploaded to EndNote X7 (Thomas Reuters) prior to the review of titles and abstracts.

Data extraction

Initial title and abstract review will be conducted by the first author (JS). Duplicates and articles clearly not meeting the selection criteria will be removed. The reference lists from identified letters to the editor, meta-analyses, case reports and reviews will be scanned to

For peer review only

identify further trials. Two-independent researchers (JS and AMV) will then read the full text of remaining relevant papers for eligibility. In cases where the two researchers cannot agree on eligibility, a third researcher will mediate. Authors of grey literature will be contacted when conference abstracts and proceedings are found. A PRISMA flowchart will be used to provide transparency of the number of papers included or excluded at each stage. Two-independent researchers (JS and AMV) will extract the data. The data extracted from the studies (if available) will include (1) authors; (2) publication year; (3) country; (4) funding; (5) setting; (6) study design; (7) sample size; (8) dosage; (9) duration of monitoring or intervention; (10) withdrawals; (11) mean age; (12) gender; (13) muscle mass; (14) physical activity; (15) muscle strength; (16) walking speed; (17) weight; (18) handgrip strength and (19) biomarkers;

Risk of bias assessment

Reporting bias will be assessed by plotting the inverse of the SEs of the effect estimates using funnel plots where meta-analysis includes more than 10 trials and will be assessed visually for asymmetry[39] and with the Egger's regression test for continuous variables.[40] Analysis will be conducted on Review Manager Software.[41]

JS and AMV will independently assess the risk of study bias using the Cochrane Collaboration's tool for assessing risk of bias in randomised trials.[42] The Cochrane risk of bias tool for RCT's consists of the following seven items: (1) random sequence generation; (2) allocation concealment; (3) blinding of participants and personnel; (4) blinding of outcome assessment; (5) incomplete outcome data; (6) selective reporting; (7) other sources of bias. Questions are rated as having a high, low or unclear level of bias across the seven domains.

Strength of evidence evaluation

Strength of evidence will be assessed by GRADE system,[43] i.e. quality of evidence for each outcome, relative importance of outcomes and overall quality of evidence.

Data management and statistical analysis

Data obtained through data extraction will be entered into Excel. Outcomes will be imported into RevMan[41] for meta-analysis. Data extracted must be presented as mean and

standard deviation, not ranges, and will not be estimated from graphs or figures. Authors will be contacted if mean and standard deviation values are not presented.

We will create a table describing study characteristics and major outcomes. We will provide a narrative synthesis of the findings from the included studies, structured around the type and content of intervention (i.e. diet alone or in combination with training), target population characteristics (i.e. sex, age, body mass index (BMI)), type of outcome (i.e. muscle strength; physical performance; muscle mass; cognitive function). We will provide summaries of intervention effects for each study by calculating risk ratios (for dichotomous outcomes) or standardised mean differences (for continuous outcomes).

We anticipate that there will be limited scope for meta-analysis because of the range of different outcomes measured across the small number of existing trials. However, where studies have used the same type of intervention, with the same outcome measure, we will pool the results using a random-effects meta-analysis, with standardised mean differences for continuous outcomes and risk ratios for binary outcomes, and calculate 95% confidence intervals and two-sided P values for each outcome. In studies where the effects of clustering have not been taken into account, we will adjust the standard deviations for the design effect. Heterogeneity between the studies in effect measures will be assessed using the I^2 statistic.[44] We will consider an I^2 value greater than 50% indicative of moderate heterogeneity or 75% high heterogeneity.[45]

We will conduct sensitivity analyses based on study quality. We will use stratified meta-analyses to explore heterogeneity in effect estimates according to participant characteristics (e.g. sex, age, BMI), location (e.g. hospital or community setting), intervention components (e.g. diet alone or in combination with training), the logistics of intervention provision.

Outcomes and prioritisation.

The primary outcomes will include recognised frailty criteria of physical activity levels; walking speed; hand-grip strength or muscle strength; and weight[4] along with changes in muscle mass and circulating levels of the pro-inflammatory markers CRP, IL-6 and TNF-α. Other outcomes will be analysed if available including body fat mass.

Patient and Public Involvement

The research question was developed following a patient involvement event in May 2017 with members from Arthritis Research UK Pain Centre and National Institute for Health Research Biomedical Research Centre Musculoskeletal PPI group. Members of the PPI group informed the authors that they already take a number of pharmaceutical treatments and they have a preference for learning more about possible lifestyle interventions, such as dietary modification where they can take control of their only health and well-being. Patients were not involved in the design of this systematic review.

ETHICS AND DISSEMINATION

No research ethics approval is required for this systematic review as no confidential patient data will be used. It is intended that the results of this systematic review will be disseminated through publication in an open access peer-reviewed journal and through conference presentations. All amendments to the protocol will be documented and dated and reported in the PROSPERO trial registry.

DISCUSSION

This systematic review will utilise rigorous methodology, to identify and examine studies reporting the outcome of omega-3 supplementation on frailty related traits on healthy ageing groups, including both inflammatory biomarkers and functional measures. No systematic review has previously addressed this objective although numerous published reviews have focused on ageing populations suffering from chronic or acute conditions. For example, there is evidence of beneficial effects of omega-3 supplementation for individuals undergoing chemotherapy or radiotherapy for cancer[46] for risk reduction in individuals with established atherosclerotic cardiovascular disease[47] and some beneficial effect on liver function in individuals with non-alcoholic fatty liver disease[48] among others.

Although risk of bias and overall level of evidence may limit analyses and confidence in this review's conclusions, this synthesis will provide a better understanding of the effect of omega-3 supplementation in preventing systemic inflammation and functional decline in the healthy elderly population.

For peer review only - http://bmjopen.bmj.com/site/about/guidelines.xhtml

Implications of results

This review will provide the first rigorous summary of effect of omega-3 supplementation across all published randomized controlled trial studies of healthy elderly individuals. The findings will inform our understanding of the value of this popular nutritional supplement in preventing frailty-related outcomes.

REFERENCES

1. United Nations D. World Population Prospects: The 2017 Revision, Key Findings and Advance Tables. Department of Economic and Social Affairs, Population Division 2017.
2. Patel HP, Syddall HE, Jameson K, et al. Prevalence of sarcopenia in community-dwelling older people in the UK using the European Working Group on Sarcopenia in Older People (EWGSOP) definition: findings from the Hertfordshire Cohort Study (HCS). *Age Ageing* 2013;42(3):378-84. doi: 10.1093/ageing/afs197
3. Davies B, Garcia F, Ara I, et al. Relationship Between Sarcopenia and Frailty in the Toledo Study of Healthy Aging: A Population Based Cross-Sectional Study. *J Am Med Dir Assoc* 2017 doi: 10.1016/j.jamda.2017.09.014
4. Fried LP, Young Y, Rubin G, et al. Self-reported preclinical disability identifies older women with early declines in performance and early disease. *J Clin Epidemiol* 2001;54(9):889-901.
5. Morley JE, Baumgartner RN, Roubenoff R, et al. Sarcopenia. *J Lab Clin Med* 2001;137(4):231-43. doi: 10.1067/mlc.2001.113504
6. Cruz-Jentoft AJ, Baeyens JP, Bauer JM, et al. Sarcopenia: European consensus on definition and diagnosis: Report of the European Working Group on Sarcopenia in Older People. *Age Ageing* 2010;39(4):412-23. doi: 10.1093/ageing/afq034
7. Balogun S, Winzenberg T, Wills K, et al. Prospective Associations of Low Muscle Mass and Function with 10-Year Falls Risk, Incident Fracture and Mortality in Community-Dwelling Older Adults. *J Nutr Health Aging* 2017;21(7):843-48. doi: 10.1007/s12603-016-0843-6
8. Leng SX, Xue QL, Tian J, et al. Inflammation and frailty in older women. *J Am Geriatr Soc* 2007;55(6):864-71. doi: 10.1111/j.1532-5415.2007.01186.x
9. Soysal P, Stubbs B, Lucato P, et al. Inflammation and frailty in the elderly: A systematic review and meta-analysis. *Ageing Res Rev* 2016;31:1-8. doi: 10.1016/j.arr.2016.08.006
10. Lorenzo-Lopez L, Maseda A, de Labra C, et al. Nutritional determinants of frailty in older adults: A systematic review. *BMC Geriatr* 2017;17(1):108. doi: 10.1186/s12877-017-0496-2
11. Puts MTE, Toubasi S, Andrew MK, et al. Interventions to prevent or reduce the level of frailty in community-dwelling older adults: a scoping review of the literature and international policies. *Age Ageing* 2017;46(3):383-92. doi: 10.1093/ageing/afw247
12. Wilkinson DJ, Bukhari SSI, Phillips BE, et al. Effects of leucine-enriched essential amino acid and whey protein bolus dosing upon skeletal muscle protein synthesis at rest and after exercise in older women. *Clin Nutr* 2017 doi: 10.1016/j.clnu.2017.09.008
13. Englund DA, Kirn DR, Koochek A, et al. Nutritional Supplementation With Physical Activity Improves Muscle Composition in Mobility-Limited Older Adults, The VIVE2 Study: A Randomized, Double-Blind, Placebo-Controlled Trial. *J Gerontol A Biol Sci Med Sci* 2017 doi: 10.1093/gerona/glx141
14. Pappalardo G, Almeida A, Ravasco P. Eicosapentaenoic acid in cancer improves body composition and modulates metabolism. *Nutrition* 2015;31(4):549-55. doi: 10.1016/j.nut.2014.12.002

For peer review only

15. Wong TC, Chen YT, Wu PY, et al. Ratio of Dietary n-6/n-3 Polyunsaturated Fatty Acids Independently Related to Muscle Mass Decline in Hemodialysis Patients. *PLoS One* 2015;10(10):e0140402. doi: 10.1371/journal.pone.0140402
16. van de Bool C, Rutten EPA, van Helvoort A, et al. A randomized clinical trial investigating the efficacy of targeted nutrition as adjunct to exercise training in COPD. *J Cachexia Sarcopenia Muscle* 2017;8(5):748-58. doi: 10.1002/jcsm.12219
17. Berbert AA, Kondo CR, Almendra CL, et al. Supplementation of fish oil and olive oil in patients with rheumatoid arthritis. *Nutrition* 2005;21(2):131-6. doi:
18. Ticinesi A, Meschi T, Lauretani F, et al. Nutrition and Inflammation in Older Individuals: Focus on Vitamin D, n-3 Polyunsaturated Fatty Acids and Whey Proteins. *Nutrients* 2016;8(4):186. doi: 10.3390/nu8040186
19. Rodriguez-Cruz M, Cruz-Guzman ODR, Almeida-Becerril T, et al. Potential therapeutic impact of omega-3 long chain-polyunsaturated fatty acids on inflammation markers in Duchenne muscular dystrophy: A double-blind, controlled randomized trial. *Clin Nutr* 2017 doi: 10.1016/j.clnu.2017.09.011
20. Yao J, Lu Y, Zhi M, et al. Dietary n3 polyunsaturated fatty acids ameliorate Crohn's disease in rats by modulating the expression of PPARgamma/NFAT. *Molecular medicine reports* 2017 doi: 10.3892/mmr.2017.7673 [published Online First: 2017/10/11]
21. Wang C, Liu W, Yao L, et al. Hydroxyeicosapentaenoic acids and epoxyeicosatetraenoic acids attenuate early occurrence of nonalcoholic fatty liver disease. *Br J Pharmacol* 2017;174(14):2358-72. doi: 10.1111/bph.13844
22. Siddiqui RA, Harvey KA, Ruzmetov N, et al. n-3 fatty acids prevent whereas trans-fatty acids induce vascular inflammation and sudden cardiac death. *Br J Nutr* 2009;102(12):1811-9. doi: 10.1017/S0007114509992030
23. Chung H, Lee YS, Mayoral R, et al. Omega-3 fatty acids reduce obesity-induced tumor progression independent of GPR120 in a mouse model of postmenopausal breast cancer. *Oncogene* 2015;34(27):3504-13. doi: 10.1038/onc.2014.283
24. Miccadei S, Masella R, Mileo AM, et al. omega3 Polyunsaturated Fatty Acids as Immunomodulators in Colorectal Cancer: New Potential Role in Adjuvant Therapies. *Front Immunol* 2016;7:486. doi: 10.3389/fimmu.2016.00486
25. Finocchiaro C, Segre O, Fadda M, et al. Effect of n-3 fatty acids on patients with advanced lung cancer: a double-blind, placebo-controlled study. *Br J Nutr* 2012;108(2):327-33. doi: 10.1017/S0007114511005551
26. Coto Montes A, Boga JA, Bermejo Millo C, et al. Potential early biomarkers of sarcopenia among independent older adults. *Maturitas* 2017;104:117-22. doi: 10.1016/j.maturitas.2017.08.009
27. Hsu B, Hirani V, Cumming RG, et al. Cross-Sectional and Longitudinal Relationships Between Inflammatory Biomarkers and Frailty in Community-dwelling Older Men: The Concord Health and Ageing in Men Project. *J Gerontol A Biol Sci Med Sci* 2017 doi: 10.1093/gerona/glx142
28. Hida T, Imagama S, Ando K, et al. Sarcopenia and physical function are associated with inflammation and arteriosclerosis in community-dwelling people: The Yakumo study. *Mod Rheumatol* 2017:1-6. doi: 10.1080/14397595.2017.1349058
29. Hutchins-Wiese H, Kleppinger A, Annis K, et al. The impact of supplemental n-3 long chain polyunsaturated fatty acids and dietary antioxidants on physical performance in postmenopausal women. *J Nutr Health Aging* 2013; 17(1).
30. Valdes AM, Ravipati S, Menni C, et al. Association of the resolvin precursor 17-HDHA, but not D- or E- series resolvins, with heat pain sensitivity and osteoarthritis pain in humans. *Sci Rep* 2017;7(1):10748. doi: 10.1038/s41598-017-09516-3
31. Di Girolamo FG, Situlin R, Mazzucco S, et al. Omega-3 fatty acids and protein metabolism: enhancement of anabolic interventions for sarcopenia. *Curr Opin Clin Nutr Metab Care* 2014;17(2):145-50. doi: 10.1097/MCO.0000000000000032

For peer review only - http://bmjopen.bmj.com/site/about/guidelines.xhtml

32. Jeromson S, Gallagher IJ, Galloway SD, et al. Omega-3 Fatty Acids and Skeletal Muscle Health. *Mar Drugs* 2015;13(11):6977-7004. doi: 10.3390/md13116977
33. Shivappa N, Stubbs B, Hebert JR, et al. The Relationship Between the Dietary Inflammatory Index and Incident Frailty: A Longitudinal Cohort Study. *J Am Med Dir Assoc* 2017 doi: 10.1016/j.jamda.2017.08.006
34. Stocks J, Valdes AM. Effect of dietary omega-3 fatty acid supplementation on frailty related phenotypes in older adults: a systematic review and meta-analysis. PROSPERO CRD42017080240. 2017.
35. Moher D, Liberati A, Tetzlaff J, et al. Preferred reporting items for systematic reviews and meta-analyses: the PRISMA statement. *J Clin Epidemiol* 2009;62(10):1006-12. doi: 10.1016/j.jclinepi.2009.06.005
36. Moher D, Shamseer L, Clarke M, et al. Preferred reporting items for systematic review and meta-analysis protocols (PRISMA-P) 2015 statement. *Syst Rev* 2015;4:1. doi: 10.1186/2046-4053-4-1
37. Shamseer L, Moher D, Clarke M, et al. Preferred reporting items for systematic review and meta-analysis protocols (PRISMA-P) 2015: elaboration and explanation. *BMJ* 2015;349:g7647. doi: 10.1136/bmj.g7647
38. Lefebvre C ME, Glanville J. Searching for studies. In: Higgins J GS, ed. Cochrane Handbook for Systematic Reviews of Interventions Version 510 The Cochrane Collaboration, 2011.
39. Sutton AJ, Duval SJ, Tweedie RL, et al. Empirical assessment of effect of publication bias on meta-analyses. *Brit Med J* 2000;320(7249):1574-77. doi: DOI 10.1136/bmj.320.7249.1574
40. Egger M, Davey Smith G, Schneider M, et al. Bias in meta-analysis detected by a simple, graphical test. *BMJ* 1997;315(7109):629-34.
41. Review Manager (RevMan) [program]. Version 5.3 version. Copenhagen: The Nordic Cochrane Centre, : The Cochrane Collaboration, 2014.
42. Higgins JP, Altman DG, Gotzsche PC, et al. The Cochrane Collaboration's tool for assessing risk of bias in randomised trials. *BMJ* 2011;343:d5928. doi: 10.1136/bmj.d5928
43. Atkins D, Best D, Briss PA, et al. Grading quality of evidence and strength of recommendations. *Bmj* 2004;328(7454):1490. doi: 10.1136/bmj.328.7454.1490
44. Higgins JP, Thompson SG. Quantifying heterogeneity in a meta-analysis. *Stat Med* 2002;21(11):1539-58. doi: 10.1002/sim.1186
45. Higgins JP, Thompson SG, Spiegelhalter DJ. A re-evaluation of random-effects meta-analysis. *J R Stat Soc Ser A Stat Soc* 2009;172(1):137-59. doi: 10.1111/j.1467-985X.2008.00552.x
46. de Aguiar Pastore Silva J, Emilia de Souza Fabre M, Waitzberg DL. Omega-3 supplements for patients in chemotherapy and/or radiotherapy: A systematic review. *Clin Nutr* 2015;34(3):359-66. doi: 10.1016/j.clnu.2014.11.005
47. Burke MF, Burke FM, Soffer DE. Review of Cardiometabolic Effects of Prescription Omega-3 Fatty Acids. *Curr Atheroscler Rep* 2017;19(12):60. doi: 10.1007/s11883-017-0700-z
48. Yu L, Yuan M, Wang L. The effect of omega-3 unsaturated fatty acids on non-alcoholic fatty liver disease: A systematic review and meta-analysis of RCTs. *Pak J Med Sci* 2017;33(4):1022-28. doi: 10.12669/pjms.334.12315

Authors' contributions: JS, the guarantor of the protocol, drafted the protocol and registered it in PROSPERO. JS and AV drafted the manuscript, contributed to the development of the selection criteria, the risk of bias assessment strategy, data extraction criteria and search strategy. All authors read, provided feedback and approved the final manuscript.

For peer review only

Acknowledgement: The authors thank Professor Jo Leonardi-Bee for her critical review of the manuscript.

Funding statement: No funding has been received for this study.

Competing interests statement: None declared

For peer review only

Supplementary File: Search Strategy

Study population terms:

- Population, target condition and outcomes: Older people and frailty

(‘aged’ OR ‘old’ OR ‘age-old’ OR ‘elder’ OR ‘senior’ OR ‘functionally impaired’ OR ‘frail’ OR ‘exp frail elderly’ OR ‘ageing’ OR ‘aging’ OR ‘post-menopausal’ OR ‘postmenopaus*’) OR ‘sarcopenia’ OR ‘hand strength’ OR ‘weight’ OR ‘walking speed’ OR ‘muscle strength’ OR ‘physical activity’).

AND

- Intervention: Omega-3 polyunsaturated fatty acid

(‘Eicosapentaenoic Acid’ OR ‘Docosahexaenoic Acid’ OR ‘Fatty Acids, Omega-3’ OR ‘Fatty Acids, Unsaturated’ OR ‘omega-3 fatty acid*’ OR ‘polyunsaturated fatty acid*’ OR ‘EPA’ OR ‘DHA’ OR ‘PUFA’ OR ‘omega-3’).

AND

- Methodology: Randomised Control Trials

(‘randomised controlled trial’ OR ‘controlled clinical trial’ OR ‘randomised’ OR ‘placebo’ OR ‘clinical trials as topic’ OR ‘randomly’ OR ’trial’)

AND

- Humans: NOT ‘animals/NOT humans’

For peer review only

1	aged.mp. or Aged/
2	old.mp.
3	age-old.mp.
4	elder.mp.
5	senior.mp.
6	Functionally-Impaired.mp.
7	frail.mp.
8	exp Frail Elderly/
9	ageing.mp.
10	Aging/ or aging.mp.
11	post-menopausal.mp.
12	postmenopaus*.mp.
13	sarcopenia.mp. or Sarcopenia/
14	hand strength/ or handgrip strength.mp.
15	weight.mp.
16	walking speed.mp. or Walking Speed/
17	muscle strength.mp. or Muscle Strength/
18	physical activity.mp. or Exercise/
19	1 or 2 or 3 or 4 or 5 or 6 or 7 or 8 or 9 or 10 or 11 or 12 or 13 or 14 or 15 or 16 or 17 or 18
20	Eicosapentaenoic Acid.mp. or Eicosapentaenoic Acid/
21	Docosahexaenoic Acid.mp. or Docosahexaenoic Acids/
22	Fatty Acids, Omega-3.mp. or Fatty Acids, Omega-3/
23	Fatty Acids, Unsaturated.mp. or Fatty Acids, Unsaturated/
24	omega-3 fatty acid*.mp.
25	polyunsaturated fatty acid*.mp.
26	EPA.mp.
27	DHA.mp.
28	PUFA.mp.
29	omega-3.mp.
30	20 or 21 or 22 or 23 or 24 or 25 or 26 or 27 or 28 or 29
31	Randomi?ed controlled trial.mp.
32	Controlled clinical trial.mp
33	Randomised.mp
34	Placebo.mp.
35	Phase 4 clinical trial.mp.
36	Single Blind Procedure.mp.
37	Double Blind Procedure.mp.
38	Crossover Procedure.mp.
39	Clinical trials.mp.
40	Randomly.mp.
41	Trial.mp.
42	31 or 32 or 33 or 34 or 35 or 36 or 37 or 38 or 39 or 40 or 41 or 42
43	19 and 30 and 42
44	exp animals/ not humans.sh.
45	43 not 44

Box 1. Example of an advanced search strategy—MEDLINE OvidSP 1946 to 30th November 2017

Stages and etail of search strategy

For peer review only

PRISMA-P 2015 Checklist

Section/topic	#	Checklist item	Information reported Yes	Information reported No	Line number(s)
**ADMINISTRATIVE INFORMATION**					
**Title**					
Identification	1a	Identify the report as a protocol of a systematic review	☒	☐	P1, 2
Update	1b	If the protocol is for an update of a previous systematic review, identify as such	☐	☒	NA
**Registration**	2	If registered, provide the name of the registry (e.g., PROSPERO) and registration number in the Abstract	☒	☐	P2, 29
**Authors**					
Contact	3a	Provide name, institutional affiliation, and e-mail address of all protocol authors; provide physical mailing address of corresponding author	☒	☐	P1, 4-22
Contributions	3b	Describe contributions of protocol authors and identify the guarantor of the review	☒	☐	P12, 31-35
**Amendments**	4	If the protocol represents an amendment of a previously completed or published protocol, identify as such and list changes; otherwise, state plan for documenting important protocol amendments	☐	☒	NA
**Support**					
Sources	5a	Indicate sources of financial or other support for the review	☒	☐	P12, 40
Sponsor	5b	Provide name for the review funder and/or sponsor	☒	☐	P12, 40
Role of sponsor/funder	5c	Describe roles of funder(s), sponsor(s), and/or institution(s), if any, in developing the protocol	☐	☒	NA
**INTRODUCTION**					
**Rationale**	6	Describe the rationale for the review in the context of what is already known	☒	☐	P3-5, 11-6
**Objectives**	7	Provide an explicit statement of the question(s) the review will address with reference to participants, interventions, comparators, and outcomes (PICO)	☒	☐	P2, 10-16
**METHODS**					
**Eligibility criteria**	8	Specify the study characteristics (e.g., PICO, study design, setting, time frame) and report	☒	☐	P5-6, 14-11

BioMed Central
The Open Access Publisher

For peer review only

Section/topic	#	Checklist item	Information reported Yes	Information reported No	Line number(s)
		characteristics (e.g., years considered, language, publication status) to be used as criteria for eligibility for the review			
**Information sources**	9	Describe all intended information sources (e.g., electronic databases, contact with study authors, trial registers, or other grey literature sources) with planned dates of coverage	☒	☐	P6, 5 & 13-20<br>P7, 3-4
**Search strategy**	10	Present draft of search strategy to be used for at least one electronic database, including planned limits, such that it could be repeated	☒	☐	Supplementary file
STUDY RECORDS					
Data management	11a	Describe the mechanism(s) that will be used to manage records and data throughout the review	☒	☐	P6, 25<br>P7-8, 30-32<br>P8, 4
Selection process	11b	State the process that will be used for selecting studies (e.g., two independent reviewers) through each phase of the review (i.e., screening, eligibility, and inclusion in meta-analysis)	☒	☐	P6-7, 28-3
Data collection process	11c	Describe planned method of extracting data from reports (e.g., piloting forms, done independently, in duplicate), any processes for obtaining and confirming data from investigators	☒	☐	P7, 5-11<br>P7, 3-4
**Data items**	12	List and define all variables for which data will be sought (e.g., PICO items, funding sources), any pre-planned data assumptions and simplifications	☒	☐	P7, 7-11
**Outcomes and prioritization**	13	List and define all outcomes for which data will be sought, including prioritization of main and additional outcomes, with rationale	☒	☐	P8, 29-32
**Risk of bias in individual studies**	14	Describe anticipated methods for assessing risk of bias of individual studies, including whether this will be done at the outcome or study level, or both; state how this information will be used in data synthesis	☒	☐	P7, 14-24
DATA					
**Synthesis**	15a	Describe criteria under which study data will be quantitatively synthesized	☒	☐	P8, 13-14
	15b	If data are appropriate for quantitative synthesis, describe planned summary measures, methods of handling data, and methods of combining data from studies, including any planned exploration of consistency (e.g., I^2, Kendall's tau)	☒	☐	P8, 15-21

BioMed Central The Open Access Publisher

3

Section/topic	#	Checklist item	Information reported Yes	Information reported No	Line number(s)
	15c	Describe any proposed additional analyses (e.g., sensitivity or subgroup analyses, meta-regression)	☒	☐	P8, 23-26
	15d	If quantitative synthesis is not appropriate, describe the type of summary planned	☒	☐	P8, 4-8
**Meta-bias(es)**	16	Specify any planned assessment of meta-bias(es) (e.g., publication bias across studies, selective reporting within studies)	☒	☐	P7, 14-24
**Confidence in cumulative evidence**	17	Describe how the strength of the body of evidence will be assessed (e.g., GRADE)	☒	☐	P7, 27-28

For peer review only

BioMed Central The Open Access Publisher

BMJ Open

Effect of dietary omega-3 fatty acid supplementation on frailty related phenotypes in older adults: a systematic review and meta-analysis protocol.

Journal:	*BMJ Open*
Manuscript ID	bmjopen-2017-021344.R2
Article Type:	Protocol
Date Submitted by the Author:	27-Mar-2018
Complete List of Authors:	Stocks, Joanne; University of Nottingham, Academic Rheumatology<br>Valdes, A; University of Nottingham,
**Primary Subject Heading**:	Geriatric medicine
Secondary Subject Heading:	Epidemiology, Nutrition and metabolism
Keywords:	Frailty, Omega-3, Older people

SCHOLARONE™
Manuscripts

For peer review only

EFFECT OF DIETARY OMEGA-3 FATTY ACID SUPPLEMENTATION ON FRAILTY RELATED PHENOTYPES IN OLDER ADULTS: A SYSTEMATIC REVIEW AND META-ANALYSIS PROTOCOL

***Joanne Stocks[123], Ana M Valdes[123]**

Author Affiliations

[1]NIHR Nottingham BRC, UK

[2]Arthritis Research UK Pain Centre, University of Nottingham, UK

[3]Division of Rheumatology, Orthopaedics and Dermatology, School of Medicine, University of Nottingham, UK

***Corresponding author:**

Joanne Stocks,

Arthritis Research UK Pain Centre,

Academic Rheumatology,

University of Nottingham,

Clinical Sciences Building,

City Hospital,

Nottingham.

NG5 1PB

Email: Joanne.Stocks@nottingham.ac.uk

Tel: 0115 8231758

Word Count: 2186

Key Words: Frailty, Omega-3, Older adults

For peer review only

ABSTRACT

Introduction

The beneficial effect of dietary omega-3 supplementation in younger adults, or older people with acute or chronic disease is established. Knowledge is now needed about the effect in medically stable older people. The objective of this study is to examine and assess the evidence for a role of dietary omega-3 polyunsaturated fatty acid (PUFA) supplementation in older adults on (1) muscle mass and muscle strength (2) inflammatory biomarkers and (3) physical activity.

Methods and analysis

A systematic review and data synthesis will be conducted of randomised controlled trials in older people not recruited for any given disease diagnosis. Placebo controlled studies reporting interventions involving dietary supplementation of omega-3 PUFAs, eicosapentaenoic acid (EPA) and docosahexaenoic acid (DHA) will be included. Outcomes must include changes from baseline to last available follow-up for one or more of the following: muscle mass; inflammatory biomarkers; physical activity; walking speed; weight change; hand grip strength or muscle strength. Once the search strategy has been carried out, two independent researchers will assess relevant papers for eligibility. Articles up until 31st December 2017 in any language will be included. We will provide a narrative synthesis of the findings from the included studies. Studies will be grouped for meta-analysis according to the outcome(s) provided. Where studies have used the same type of intervention, with the same outcome measure, we will pool the results using a random-effects meta-analysis, with standardised mean differences for continuous outcomes and risk ratios for binary outcomes, and calculate 95% confidence intervals and two-sided P values for each outcome.

Ethics and dissemination

No research ethics approval is required for this systematic review, as no confidential patient data will be used. The results of this systematic review will be disseminated through publication in an open access peer-reviewed journal and through conference presentations.

PROSPERO registration number: CRD42017080240

For peer review only

STRENGTHS AND LIMITATIONS OF THIS STUDY

- This will be the first study to systematically examine the effect of dietary omega-3 fatty acid supplementation on frailty related phenotypes inolder adults not selected for specific chronic or acute conditions.
- An important strength of the study is the focus on both functional (walking speed, grip strength, get up and go) and inflammatory outcomes (cytokine level) outcomes which will allow to put in context the effect size and direction of effect of omega-3 supplementation and to prioritise outcomes for future RCTs.
- Results will also help to inform future guidelines on dietary supplementation for older adults.
- Limitations may include issues of poor reporting affecting risk of bias assessment and confidence in results.

INTRODUCTION

According to the United Nations, the number of people aged over 60 will double globally from 962 million in 2017 to 2.1 billion in 2050.[1] In Europe, the proportion of the population aged over 60 is projected to reach 35% by 2050.[1] It is, therefore, a global priority to ensure that this ageing population remains independent. A key element of maintaining independence in older adults is the preservation of mobility along with muscle mass and strength.

Muscle mass decline is one of the hallmarks of ageing and, from age 40 muscle mass begins to decrease, with an annual decline in functional capacity of up to 3% per year after age 60.[2] A key gerontological concept linked to musculoskeletal ageing is frailty.[3] The commonly acknowledged characteristics include unintentional weight loss, self-reported exhaustion, weakness (grip strength), slow walking speed, and low physical activity.[4] This complex phenomenon is highly correlated with loss of mobility along with progressive loss of skeletal muscle strength (dynapenia), mass and function (sarcopenia)[5] and results in a reduced quality of life and is a major public health concern.[6]

For peer review only

The prevalence of frailty also increases with age, and along with sarcopenia is associated with serious adverse outcomes, including falls, hospitalisation and mortality.[7] There is consensus of an inflammatory contribution to frailty. Striking differences in the levels of pro-inflammatory cytokines between frail and non-frail elderly have been reported,[8] and predict higher mortality.[9]

A role for nutritional determinants of frailty has been proposed[10] and a number of lifestyle interventions have been investigated with regards to frailty, including exercise and increased protein intake.[11]

Recent work has also begun to investigate such interventions to prevent or diminish muscle loss in medical settings, including the supplementation of leucine[12], vitamin D[13] and fish-derived polyunsaturated omega 3 fatty acids (PUFA), eicosapentaenoic acid (EPA) and docosahexaenoic acid (DHA). Studies carried out in a variety of populations including cancer patients,[14] patients with end-stage renal disease,[15] chronic obstructive pulmonary disease[16] and rheumatoid arthritis[17] have shown that dietary PUFAs have a beneficial effect on skeletal muscle mass and strength.

Dietary supplementation of omega-3 PUFAs is of particular interest in the context of frailty, given its well-known anti-inflammatory role and the importance of inflammation in the development of ageing.[18]

Omega-3 reduces inflammation in conditions including Duchenne muscular dystrophy,[19] Crohn's disease,[20] non-alcoholic fatty liver disease,[21] cardiovascular disease[22] as well as many cancers.[14 23-25] These studies are of particular interest, as increased levels of inflammatory biomarkers such as interleukin-6 (IL-6), tumour necrosis factor-alpha (TNFα) and C reactive protein (CRP) have all been linked with both frailty and sarcopenia in older adults.[26-28] Long chain PUFAs have been suggested to interact with antioxidants and improve inflammatory responses to positively impact on physical performance.[29] Part of the mechanism may involve the effect of omega-3 on musculoskeletal pain; a pain reduction would be conducive to more physical activity.[30] More generally, omega-3 supplementation may act directly on skeletal muscle and improve protein metabolism hence having an

For peer review only

influence on physical performance. [31 32] A more pro-inflammatory diet has also been linked with a higher incidence of frailty.[33]

A recent review by Ticinesi *et al.*[18] summarised the analysis of omega-3 PUFAs on inflammation in older individuals in both cross-sectional and randomized controlled trials. However, we are not aware of any systematic reviews or meta-analyses focussing specifically on omega-3 fatty acid supplementation on frailty phenotypes in older adults not selected for any specific chronic or acute conditions. We propose to conduct a systematic review and meta-analysis to examine the effect of dietary omega-3 supplementation in older people not recruited for any given disease diagnosis. The outcomes that will be investigated are inflammatory biomarkers; muscle mass; physical activity; walking speed; weight change; hand grip strength or muscle strength, as well as biases in the included studies.

METHODS AND ANALYSIS

Registration

This protocol has been registered with PROSPERO (registration number CRD42017080240[34]) and reported in accordance with Preferred Reporting Items for Systematic Reviews and Meta-Analyses (PRISMA)[35] and PRISMA-Protocol [36 37] guidelines.

Study selection criteria

Interventions and population

Studies reporting results of interventions involving dietary supplementation of omega-3 PUFAs will be included. Dietary supplementation will be defined as daily ingestion of capsules containing EPA and DHA or through an EPA and DHA enriched diet. The comparator will be placebo-controlled groups. Participants will include community-dwelling, persons, of either sex, classified by the study authors as postmenopausal or older people with the majority of participants over 60 years of age. To ensure the focus of the review is on older people not recruited for any given disease diagnosis, exclusions will be studies where participants were selected because they had a cancer or other chronic disease diagnosis. Participants who currently consumed a high fish diet or use fish oil supplements will also be excluded.

For peer review only

Outcomes

Outcomes must include changes from baseline to last available follow-up for one or more of the following: muscle mass; inflammatory biomarkers; physical activity; walking speed; weight change; hand grip strength or muscle strength. Any adverse effects will also be summarised. Studies will be grouped for meta-analysis according to the outcome(s) provided.

Study designs

Randomised controlled trials (RCTs) will be included.

Other

Articles up until 31st December 2017 in any language will be included.

Exclusion criteria

Studies will be excluded for the following reasons: (1) study population was specifically focused on participants diagnosed and being treated for a pre-existing medical condition (e.g. cancer, kidney disease, liver disease, diabetes mellitus, cardiovascular disease); or (2) letters to the editor, meta-analyses, case reports and reviews.

Search strategy

MEDLINE (OVID) from 1946, the Cochrane Register of Controlled Trials (CENTRAL) from 1940, EMBASE from 1946, Cumulative Index to Nursing and Allied Health Literature (CINAHL) from 1937, Allied and Complementary Medicine Database (AMED) and Web of Science will be searched for relevant trials. The search strategy for Medline has been developed in consultation with a subject-specific librarian and will be adapted for use in other databases. Search terms are informed by Cochrane Handbook[38] and other systematic reviews investigating PUFA dietary supplementation, sarcopenia and/or frailty.

The full search strategy can be found in the supplementary file 1. Example of searches that will be used can be seen in supplementary file 2, Box 1 MEDLINE (OVID) Advanced Search Example. Syntax (truncation, wildcards and quotation marks) and operators will be

For peer review only

amended according to the specific databases. Initial search results will be uploaded to EndNote X7 (Thomas Reuters) prior to the review of titles and abstracts.

Data extraction

Initial title and abstract review will be conducted by the first author (JS). Duplicates and articles clearly not meeting the selection criteria will be removed. The reference lists from identified letters to the editor, meta-analyses, case reports and reviews will be scanned to identify further trials. Two-independent researchers (JS and AMV) will then read the full text of remaining relevant papers for eligibility. In cases where the two researchers cannot agree on eligibility, a third researcher will mediate. Authors of grey literature will be contacted when conference abstracts and proceedings are found. A PRISMA flowchart will be used to provide transparency of the number of papers included or excluded at each stage. Two-independent researchers (JS and AMV) will extract the data. The data extracted from the studies (if available) will include (1) authors; (2) publication year; (3) country; (4) funding; (5) setting; (6) study design; (7) sample size; (8) dosage; (9) duration of monitoring or intervention; (10) withdrawals; (11) mean age; (12) gender; (13) muscle mass; (14) physical activity; (15) muscle strength; (16) walking speed; (17) weight; (18) handgrip strength and (19) biomarkers;

Risk of bias assessment

Reporting bias will be assessed by plotting the inverse of the SEs of the effect estimates using funnel plots where meta-analysis includes more than 10 trials and will be assessed visually for asymmetry[39] and with the Egger's regression test for continuous variables.[40] Analysis will be conducted on Review Manager Software.[41]

JS and AMV will independently assess the risk of study bias using the Cochrane Collaboration’s tool for assessing risk of bias in randomised trials.[42] The Cochrane risk of bias tool for RCT’s consists of the following seven items: (1) random sequence generation; (2) allocation concealment; (3) blinding of participants and personnel; (4) blinding of outcome assessment; (5) incomplete outcome data; (6) selective reporting; (7) other sources of bias. Questions are rated as having a high, low or unclear level of bias across the seven domains.

For peer review only

Strength of evidence evaluation

Strength of evidence will be assessed by GRADE system,[43] i.e. quality of evidence for each outcome, relative importance of outcomes and overall quality of evidence.

Data management and statistical analysis

Data obtained through data extraction will be entered into Excel. Outcomes will be imported into RevMan[41] for meta-analysis. Data extracted must be presented as mean and standard deviation, not ranges, and will not be estimated from graphs or figures. Authors will be contacted if mean and standard deviation values are not presented.

We will create a table describing study characteristics and major outcomes. We will provide a narrative synthesis of the findings from the included studies, structured around the type and content of intervention (i.e. diet alone or in combination with training), target population characteristics (i.e. sex, age, body mass index (BMI)), type of outcome (i.e. muscle strength; physical performance; muscle mass; cognitive function). We will provide summaries of intervention effects for each study by calculating risk ratios (for dichotomous outcomes) or standardised mean differences (for continuous outcomes).

We anticipate that there will be limited scope for meta-analysis because of the range of different outcomes measured across the small number of existing trials. However, where studies have used the same type of intervention, with the same outcome measure, we will pool the results using a random-effects meta-analysis, with standardised mean differences for continuous outcomes and risk ratios for binary outcomes, and calculate 95% confidence intervals and two-sided P values for each outcome. In studies where the effects of clustering have not been taken into account, we will adjust the standard deviations for the design effect. Heterogeneity between the studies in effect measures will be assessed using the I^2 statistic.[44] We will consider an I^2 value greater than 50% indicative of moderate heterogeneity or 75% high heterogeneity.[45]

We will conduct sensitivity analyses based on study quality. We will use stratified meta-analyses to explore heterogeneity in effect estimates according to participant characteristics

For peer review only - http://bmjopen.bmj.com/site/about/guidelines.xhtml

(e.g. sex, age, BMI), location (e.g. hospital or community setting), intervention components (e.g. diet alone or in combination with training), the logistics of intervention provision.

Outcomes and prioritisation.

The primary outcomes will include recognised frailty criteria of physical activity levels; walking speed; hand-grip strength or muscle strength; and weight[4] along with changes in muscle mass and circulating levels of the pro-inflammatory markers CRP, IL-6 and TNF-α. Other outcomes will be analysed if available including body fat mass.

Patient and Public Involvement

The research question was developed following a patient involvement event in May 2017 with members from Arthritis Research UK Pain Centre and National Institute for Health Research Biomedical Research Centre Musculoskeletal PPI group. Members of the PPI group informed the authors that they already take a number of pharmaceutical treatments and they have a preference for learning more about possible lifestyle interventions, such as dietary modification where they can take control of their only health and well-being. Patients were not involved in the design of this systematic review.

ETHICS AND DISSEMINATION

No research ethics approval is required for this systematic review, as no confidential patient data will be used. It is intended that the results of this systematic review will be disseminated through publication in an open access peer-reviewed journal and through conference presentations. All amendments to the protocol will be documented, dated and reported in the PROSPERO trial registry.

DISCUSSION

This systematic review will utilise rigorous methodology, to identify and examine studies reporting the outcome of omega-3 supplementation on frailty related traits on ageing groups not selected for specific chronic or acute conditions, including both inflammatory biomarkers and functional measures. No systematic review has previously addressed this objective although numerous published reviews have focused on ageing populations suffering from chronic or acute conditions. For example, there is evidence of beneficial

For peer review

effects of omega-3 supplementation for individuals undergoing chemotherapy or radiotherapy for cancer[46] for risk reduction in individuals with established atherosclerotic cardiovascular disease[47] and some beneficial effect on liver function in individuals with non-alcoholic fatty liver disease[48] among others.

Although risk of bias and overall level of evidence may limit analyses and confidence in this review's conclusions, this synthesis will provide a better understanding of the effect of omega-3 supplementation in preventing systemic inflammation and functional decline in the elderly population.

Implications of results

This review will provide the first rigorous summary of effect of omega-3 supplementation across all published randomized controlled trial studies of elderly individuals not selected for chronic or acute conditions. The findings will inform our understanding of the value of this popular nutritional supplement in preventing frailty-related outcomes.

REFERENCES

1. United Nations D. World Population Prospects: The 2017 Revision, Key Findings and Advance Tables. Department of Economic and Social Affairs, Population Division 2017.
2. Patel HP, Syddall HE, Jameson K, et al. Prevalence of sarcopenia in community-dwelling older people in the UK using the European Working Group on Sarcopenia in Older People (EWGSOP) definition: findings from the Hertfordshire Cohort Study (HCS). *Age Ageing* 2013;42(3):378-84. doi: 10.1093/ageing/afs197
3. Davies B, Garcia F, Ara I, et al. Relationship Between Sarcopenia and Frailty in the Toledo Study of Healthy Aging: A Population Based Cross-Sectional Study. *J Am Med Dir Assoc* 2017 doi: 10.1016/j.jamda.2017.09.014
4. Fried LP, Young Y, Rubin G, et al. Self-reported preclinical disability identifies older women with early declines in performance and early disease. *J Clin Epidemiol* 2001;54(9):889-901.
5. Morley JE, Baumgartner RN, Roubenoff R, et al. Sarcopenia. *J Lab Clin Med* 2001;137(4):231-43. doi: 10.1067/mlc.2001.113504
6. Cruz-Jentoft AJ, Baeyens JP, Bauer JM, et al. Sarcopenia: European consensus on definition and diagnosis: Report of the European Working Group on Sarcopenia in Older People. *Age Ageing* 2010;39(4):412-23. doi: 10.1093/ageing/afq034
7. Balogun S, Winzenberg T, Wills K, et al. Prospective Associations of Low Muscle Mass and Function with 10-Year Falls Risk, Incident Fracture and Mortality in Community-Dwelling Older Adults. *J Nutr Health Aging* 2017;21(7):843-48. doi: 10.1007/s12603-016-0843-6
8. Leng SX, Xue QL, Tian J, et al. Inflammation and frailty in older women. *J Am Geriatr Soc* 2007;55(6):864-71. doi: 10.1111/j.1532-5415.2007.01186.x
9. Soysal P, Stubbs B, Lucato P, et al. Inflammation and frailty in the elderly: A systematic review and meta-analysis. *Ageing Res Rev* 2016;31:1-8. doi: 10.1016/j.arr.2016.08.006

For peer review only

10. Lorenzo-Lopez L, Maseda A, de Labra C, et al. Nutritional determinants of frailty in older adults: A systematic review. *BMC Geriatr* 2017;17(1):108. doi: 10.1186/s12877-017-0496-2
11. Puts MTE, Toubasi S, Andrew MK, et al. Interventions to prevent or reduce the level of frailty in community-dwelling older adults: a scoping review of the literature and international policies. *Age Ageing* 2017;46(3):383-92. doi: 10.1093/ageing/afw247
12. Wilkinson DJ, Bukhari SSI, Phillips BE, et al. Effects of leucine-enriched essential amino acid and whey protein bolus dosing upon skeletal muscle protein synthesis at rest and after exercise in older women. *Clin Nutr* 2017 doi: 10.1016/j.clnu.2017.09.008
13. Englund DA, Kirn DR, Koochek A, et al. Nutritional Supplementation With Physical Activity Improves Muscle Composition in Mobility-Limited Older Adults, The VIVE2 Study: A Randomized, Double-Blind, Placebo-Controlled Trial. *J Gerontol A Biol Sci Med Sci* 2017 doi: 10.1093/gerona/glx141
14. Pappalardo G, Almeida A, Ravasco P. Eicosapentaenoic acid in cancer improves body composition and modulates metabolism. *Nutrition* 2015;31(4):549-55. doi: 10.1016/j.nut.2014.12.002
15. Wong TC, Chen YT, Wu PY, et al. Ratio of Dietary n-6/n-3 Polyunsaturated Fatty Acids Independently Related to Muscle Mass Decline in Hemodialysis Patients. *PLoS One* 2015;10(10):e0140402. doi: 10.1371/journal.pone.0140402
16. van de Bool C, Rutten EPA, van Helvoort A, et al. A randomized clinical trial investigating the efficacy of targeted nutrition as adjunct to exercise training in COPD. *J Cachexia Sarcopenia Muscle* 2017;8(5):748-58. doi: 10.1002/jcsm.12219
17. Berbert AA, Kondo CR, Almendra CL, et al. Supplementation of fish oil and olive oil in patients with rheumatoid arthritis. *Nutrition* 2005;21(2):131-6. doi:
18. Ticinesi A, Meschi T, Lauretani F, et al. Nutrition and Inflammation in Older Individuals: Focus on Vitamin D, n-3 Polyunsaturated Fatty Acids and Whey Proteins. *Nutrients* 2016;8(4):186. doi: 10.3390/nu8040186
19. Rodriguez-Cruz M, Cruz-Guzman ODR, Almeida-Becerril T, et al. Potential therapeutic impact of omega-3 long chain-polyunsaturated fatty acids on inflammation markers in Duchenne muscular dystrophy: A double-blind, controlled randomized trial. *Clin Nutr* 2017 doi: 10.1016/j.clnu.2017.09.011
20. Yao J, Lu Y, Zhi M, et al. Dietary n3 polyunsaturated fatty acids ameliorate Crohn's disease in rats by modulating the expression of PPARgamma/NFAT. *Molecular medicine reports* 2017 doi: 10.3892/mmr.2017.7673 [published Online First: 2017/10/11]
21. Wang C, Liu W, Yao L, et al. Hydroxyeicosapentaenoic acids and epoxyeicosatetraenoic acids attenuate early occurrence of nonalcoholic fatty liver disease. *Br J Pharmacol* 2017;174(14):2358-72. doi: 10.1111/bph.13844
22. Siddiqui RA, Harvey KA, Ruzmetov N, et al. n-3 fatty acids prevent whereas trans-fatty acids induce vascular inflammation and sudden cardiac death. *Br J Nutr* 2009;102(12):1811-9. doi: 10.1017/S0007114509992030
23. Chung H, Lee YS, Mayoral R, et al. Omega-3 fatty acids reduce obesity-induced tumor progression independent of GPR120 in a mouse model of postmenopausal breast cancer. *Oncogene* 2015;34(27):3504-13. doi: 10.1038/onc.2014.283
24. Miccadei S, Masella R, Mileo AM, et al. omega3 Polyunsaturated Fatty Acids as Immunomodulators in Colorectal Cancer: New Potential Role in Adjuvant Therapies. *Front Immunol* 2016;7:486. doi: 10.3389/fimmu.2016.00486
25. Finocchiaro C, Segre O, Fadda M, et al. Effect of n-3 fatty acids on patients with advanced lung cancer: a double-blind, placebo-controlled study. *Br J Nutr* 2012;108(2):327-33. doi: 10.1017/S0007114511005551
26. Coto Montes A, Boga JA, Bermejo Millo C, et al. Potential early biomarkers of sarcopenia among independent older adults. *Maturitas* 2017;104:117-22. doi: 10.1016/j.maturitas.2017.08.009

For peer review only

27. Hsu B, Hirani V, Cumming RG, et al. Cross-Sectional and Longitudinal Relationships Between Inflammatory Biomarkers and Frailty in Community-dwelling Older Men: The Concord Health and Ageing in Men Project. *J Gerontol A Biol Sci Med Sci* 2017 doi: 10.1093/gerona/glx142
28. Hida T, Imagama S, Ando K, et al. Sarcopenia and physical function are associated with inflammation and arteriosclerosis in community-dwelling people: The Yakumo study. *Mod Rheumatol* 2017:1-6. doi: 10.1080/14397595.2017.1349058
29. Hutchins-Wiese H, Kleppinger A, Annis K, et al. The impact of supplemental n-3 long chain polyunsaturated fatty acids and dietary antioxidants on physical performance in postmenopausal women. *J Nutr Health Aging* 2013; 17(1).
30. Valdes AM, Ravipati S, Menni C, et al. Association of the resolvin precursor 17-HDHA, but not D- or E- series resolvins, with heat pain sensitivity and osteoarthritis pain in humans. *Sci Rep* 2017;7(1):10748. doi: 10.1038/s41598-017-09516-3
31. Di Girolamo FG, Situlin R, Mazzucco S, et al. Omega-3 fatty acids and protein metabolism: enhancement of anabolic interventions for sarcopenia. *Curr Opin Clin Nutr Metab Care* 2014;17(2):145-50. doi: 10.1097/MCO.0000000000000032
32. Jeromson S, Gallagher IJ, Galloway SD, et al. Omega-3 Fatty Acids and Skeletal Muscle Health. *Mar Drugs* 2015;13(11):6977-7004. doi: 10.3390/md13116977
33. Shivappa N, Stubbs B, Hebert JR, et al. The Relationship Between the Dietary Inflammatory Index and Incident Frailty: A Longitudinal Cohort Study. *J Am Med Dir Assoc* 2017 doi: 10.1016/j.jamda.2017.08.006
34. Stocks J, Valdes AM. Effect of dietary omega-3 fatty acid supplementation on frailty related phenotypes in older adults: a systematic review and meta-analysis. PROSPERO CRD42017080240. 2017.
35. Moher D, Liberati A, Tetzlaff J, et al. Preferred reporting items for systematic reviews and meta-analyses: the PRISMA statement. *J Clin Epidemiol* 2009;62(10):1006-12. doi: 10.1016/j.jclinepi.2009.06.005
36. Moher D, Shamseer L, Clarke M, et al. Preferred reporting items for systematic review and meta-analysis protocols (PRISMA-P) 2015 statement. *Syst Rev* 2015;4:1. doi: 10.1186/2046-4053-4-1
37. Shamseer L, Moher D, Clarke M, et al. Preferred reporting items for systematic review and meta-analysis protocols (PRISMA-P) 2015: elaboration and explanation. *BMJ* 2015;349:g7647. doi: 10.1136/bmj.g7647
38. Lefebvre C ME, Glanville J. Searching for studies. In: Higgins J GS, ed. Cochrane Handbook for Systematic Reviews of Interventions Version 510 The Cochrane Collaboration, 2011.
39. Sutton AJ, Duval SJ, Tweedie RL, et al. Empirical assessment of effect of publication bias on meta-analyses. *Brit Med J* 2000;320(7249):1574-77. doi: DOI 10.1136/bmj.320.7249.1574
40. Egger M, Davey Smith G, Schneider M, et al. Bias in meta-analysis detected by a simple, graphical test. *BMJ* 1997;315(7109):629-34.
41. Review Manager (RevMan) [program]. Version 5.3 version. Copenhagen: The Nordic Cochrane Centre, : The Cochrane Collaboration, 2014.
42. Higgins JP, Altman DG, Gotzsche PC, et al. The Cochrane Collaboration's tool for assessing risk of bias in randomised trials. *BMJ* 2011;343:d5928. doi: 10.1136/bmj.d5928
43. Atkins D, Best D, Briss PA, et al. Grading quality of evidence and strength of recommendations. *Bmj* 2004;328(7454):1490. doi: 10.1136/bmj.328.7454.1490
44. Higgins JP, Thompson SG. Quantifying heterogeneity in a meta-analysis. *Stat Med* 2002;21(11):1539-58. doi: 10.1002/sim.1186
45. Higgins JP, Thompson SG, Spiegelhalter DJ. A re-evaluation of random-effects meta-analysis. *J R Stat Soc Ser A Stat Soc* 2009;172(1):137-59. doi: 10.1111/j.1467-985X.2008.00552.x
46. de Aguiar Pastore Silva J, Emilia de Souza Fabre M, Waitzberg DL. Omega-3 supplements for patients in chemotherapy and/or radiotherapy: A systematic review. *Clin Nutr* 2015;34(3):359-66. doi: 10.1016/j.clnu.2014.11.005

For peer review only

47. Burke MF, Burke FM, Soffer DE. Review of Cardiometabolic Effects of Prescription Omega-3 Fatty Acids. *Curr Atheroscler Rep* 2017;19(12):60. doi: 10.1007/s11883-017-0700-z
48. Yu L, Yuan M, Wang L. The effect of omega-3 unsaturated fatty acids on non-alcoholic fatty liver disease: A systematic review and meta-analysis of RCTs. *Pak J Med Sci* 2017;33(4):1022-28. doi: 10.12669/pjms.334.12315

Authors' contributions: JS, the guarantor of the protocol, drafted the protocol and registered it in PROSPERO. JS and AV drafted the manuscript, contributed to the development of the selection criteria, the risk of bias assessment strategy, data extraction criteria and search strategy. All authors read, provided feedback and approved the final manuscript.

Acknowledgement: The authors thank Professor Jo Leonardi-Bee for her critical review of the manuscript.

Funding statement: No funding has been received for this study.

Competing interests statement: None declared

For peer review only

Supplementary File: Search Strategy

Study population terms:

- Population, target condition and outcomes: Older people and frailty

('aged' OR 'old' OR 'age-old' OR 'elder' OR 'senior' OR 'functionally impaired' OR 'frail' OR 'exp frail elderly' OR 'ageing' OR 'aging' OR 'post-menopausal' OR 'postmenopaus*') OR 'sarcopenia' OR 'hand strength' OR 'weight' OR 'walking speed' OR 'muscle strength' OR 'physical activity').

AND

- Intervention: Omega-3 polyunsaturated fatty acid

('Eicosapentaenoic Acid' OR 'Docosahexaenoic Acid' OR 'Fatty Acids, Omega-3' OR 'Fatty Acids, Unsaturated' OR 'omega-3 fatty acid*' OR 'polyunsaturated fatty acid*' OR 'EPA' OR 'DHA' OR 'PUFA' OR 'omega-3').

AND

- Methodology: Randomised Control Trials

('randomised controlled trial' OR 'controlled clinical trial' OR 'randomised' OR 'placebo' OR 'clinical trials as topic' OR 'randomly' OR 'trial')

AND

- Humans: NOT 'animals/NOT humans'

For peer review only

1	aged.mp. or Aged/
2	old.mp.
3	age-old.mp.
4	elder.mp.
5	senior.mp.
6	Functionally-Impaired.mp.
7	frail.mp.
8	exp Frail Elderly/
9	ageing.mp.
10	Aging/ or aging.mp.
11	post-menopausal.mp.
12	postmenopaus*.mp.
13	sarcopenia.mp. or Sarcopenia/
14	hand strength/ or handgrip strength.mp.
15	weight.mp.
16	walking speed.mp. or Walking Speed/
17	muscle strength.mp. or Muscle Strength/
18	physical activity.mp. or Exercise/
19	1 or 2 or 3 or 4 or 5 or 6 or 7 or 8 or 9 or 10 or 11 or 12 or 13 or 14 or 15 or 16 or 17 or 18
20	Eicosapentaenoic Acid.mp. or Eicosapentaenoic Acid/
21	Docosahexaenoic Acid.mp. or Docosahexaenoic Acids/
22	Fatty Acids, Omega-3.mp. or Fatty Acids, Omega-3/
23	Fatty Acids, Unsaturated.mp. or Fatty Acids, Unsaturated/
24	omega-3 fatty acid*.mp.
25	polyunsaturated fatty acid*.mp.
26	EPA.mp.
27	DHA.mp.
28	PUFA.mp.
29	omega-3.mp.
30	20 or 21 or 22 or 23 or 24 or 25 or 26 or 27 or 28 or 29
31	Randomi?ed controlled trial.mp.
32	Controlled clinical trial.mp
33	Randomised.mp
34	Placebo.mp.
35	Phase 4 clinical trial.mp.
36	Single Blind Procedure.mp.
37	Double Blind Procedure.mp.
38	Crossover Procedure.mp.
39	Clinical trials.mp.
40	Randomly.mp.
41	Trial.mp.
42	31 or 32 or 33 or 34 or 35 or 36 or 37 or 38 or 39 or 40 or 41 or 42
43	19 and 30 and 42
44	exp animals/ not humans.sh.
45	43 not 44

Box 1. Example of an advanced search strategy—MEDLINE OvidSP 1946 to 30th November 2017

Stages and detail of search strategy

For peer review only

PRISMA-P 2015 Checklist

Section/topic	#	Checklist item	Information reported Yes	Information reported No	Line number(s)
**ADMINISTRATIVE INFORMATION**					
**Title**					
Identification	1a	Identify the report as a protocol of a systematic review	☒	☐	P1, 2
Update	1b	If the protocol is for an update of a previous systematic review, identify as such	☐	☒	NA
**Registration**	2	If registered, provide the name of the registry (e.g., PROSPERO) and registration number in the Abstract	☒	☐	P2, 29
**Authors**					
Contact	3a	Provide name, institutional affiliation, and e-mail address of all protocol authors; provide physical mailing address of corresponding author	☒	☐	P1, 4-22
Contributions	3b	Describe contributions of protocol authors and identify the guarantor of the review	☒	☐	P12, 31-35
**Amendments**	4	If the protocol represents an amendment of a previously completed or published protocol, identify as such and list changes; otherwise, state plan for documenting important protocol amendments	☐	☒	NA
**Support**					
Sources	5a	Indicate sources of financial or other support for the review	☒	☐	P12, 40
Sponsor	5b	Provide name for the review funder and/or sponsor	☒	☐	P12, 40
Role of sponsor/funder	5c	Describe roles of funder(s), sponsor(s), and/or institution(s), if any, in developing the protocol	☐	☒	NA
**INTRODUCTION**					
**Rationale**	6	Describe the rationale for the review in the context of what is already known	☒	☐	P3-5, 11-6
**Objectives**	7	Provide an explicit statement of the question(s) the review will address with reference to participants, interventions, comparators, and outcomes (PICO)	☒	☐	P2, 10-16
**METHODS**					
**Eligibility criteria**	8	Specify the study characteristics (e.g., PICO, study design, setting, time frame) and report	☒	☐	P5-6, 14-11

For peer review only

BioMed Central
The Open Access Publisher

Section/topic	#	Checklist item	Information reported		Line number(s)
			Yes	No	
		characteristics (e.g., years considered, language, publication status) to be used as criteria for eligibility for the review			
**Information sources**	9	Describe all intended information sources (e.g., electronic databases, contact with study authors, trial registers, or other grey literature sources) with planned dates of coverage	☒	☐	P6, 5 & 13-20<br>P7, 3-4
**Search strategy**	10	Present draft of search strategy to be used for at least one electronic database, including planned limits, such that it could be repeated	☒	☐	Supplementary file
STUDY RECORDS					
Data management	11a	Describe the mechanism(s) that will be used to manage records and data throughout the review	☒	☐	P6, 25<br>P7-8, 30-32<br>P8, 4
Selection process	11b	State the process that will be used for selecting studies (e.g., two independent reviewers) through each phase of the review (i.e., screening, eligibility, and inclusion in meta-analysis)	☒	☐	P6-7, 28-3
Data collection process	11c	Describe planned method of extracting data from reports (e.g., piloting forms, done independently, in duplicate), any processes for obtaining and confirming data from investigators	☒	☐	P7, 5-11<br>P7, 3-4
**Data items**	12	List and define all variables for which data will be sought (e.g., PICO items, funding sources), any pre-planned data assumptions and simplifications	☒	☐	P7, 7-11
**Outcomes and prioritization**	13	List and define all outcomes for which data will be sought, including prioritization of main and additional outcomes, with rationale	☒	☐	P8, 29-32
**Risk of bias in individual studies**	14	Describe anticipated methods for assessing risk of bias of individual studies, including whether this will be done at the outcome or study level, or both; state how this information will be used in data synthesis	☒	☐	P7, 14-24
DATA					
**Synthesis**	15a	Describe criteria under which study data will be quantitatively synthesized	☒	☐	P8, 13-14
	15b	If data are appropriate for quantitative synthesis, describe planned summary measures, methods of handling data, and methods of combining data from studies, including any planned exploration of consistency (e.g., I^2, Kendall's tau)	☒	☐	P8, 15-21

BioMed Central
The Open Access Publisher

3

Section/topic	#	Checklist item	Information reported Yes	No	Line number(s)
	15c	Describe any proposed additional analyses (e.g., sensitivity or subgroup analyses, meta-regression)	☒	☐	P8, 23-26
	15d	If quantitative synthesis is not appropriate, describe the type of summary planned	☒	☐	P8, 4-8
**Meta-bias(es)**	16	Specify any planned assessment of meta-bias(es) (e.g., publication bias across studies, selective reporting within studies)	☒	☐	P7, 14-24
**Confidence in cumulative evidence**	17	Describe how the strength of the body of evidence will be assessed (e.g., GRADE)	☒	☐	P7, 27-28

For peer review only

BioMed Central The Open Access Publisher
